# PolyQ-Expansion Causes Mitochondria Fragmentation Independent of Huntingtin and Is Distinct from Traumatic Brain Injury (TBI)/Mechanical Stress-Mediated Fragmentation Which Results from Cell Death

**DOI:** 10.3390/cells12192406

**Published:** 2023-10-05

**Authors:** Kelsey Swinter, Dania Salah, Rasika Rathnayake, Shermali Gunawardena

**Affiliations:** Department of Biological Sciences, State University of New York at Buffalo, Buffalo, NY 14260, USA

**Keywords:** huntingtin, expansion of polyQ repeats, *Drosophila*, mechanical stress/traumatic brain injury, mitochondria

## Abstract

Mitochondrial dysfunction has been reported in many Huntington’s disease (HD) models; however, it is unclear how these defects occur. Here, we test the hypothesis that excess pathogenic huntingtin (HTT) impairs mitochondrial homeostasis, using *Drosophila* genetics and pharmacological inhibitors in HD and polyQ-expansion disease models and in a mechanical stress-induced traumatic brain injury (TBI) model. Expression of pathogenic HTT caused fragmented mitochondria compared to normal HTT, but HTT did not co-localize with mitochondria under normal or pathogenic conditions. Expression of pathogenic polyQ (127Q) alone or in the context of Machado Joseph Disease (MJD) caused fragmented mitochondria. While mitochondrial fragmentation was not dependent on the cellular location of polyQ accumulations, the expression of a chaperone protein, excess of mitofusin (MFN), or depletion of dynamin-related protein 1 (DRP1) rescued fragmentation. Intriguingly, a higher concentration of nitric oxide (NO) was observed in polyQ-expressing larval brains and inhibiting NO production rescued polyQ-mediated fragmented mitochondria, postulating that DRP1 nitrosylation could contribute to excess fission. Furthermore, while excess PI3K, which suppresses polyQ-induced cell death, did not rescue polyQ-mediated fragmentation, it did rescue fragmentation caused by mechanical stress/TBI. Together, our observations suggest that pathogenic polyQ alone is sufficient to cause DRP1-dependent mitochondrial fragmentation upstream of cell death, uncovering distinct physiological mechanisms for mitochondrial dysfunction in polyQ disease and mechanical stress.

## 1. Introduction

Mitochondria are the main energy source for cells, and as such, proper maintenance of mitochondrial health is critical for cell survival. Impaired mitochondria homeostasis can have disastrous consequences, contributing to cell death, including those seen in many neurodegenerative diseases. Mitochondrial homeostasis involves the balance between several different processes, including fission, fusion, mitophagy and transport [1]. Mitochondria fission, also known as biogenesis, involves the division of one mitochondrion into two daughter organelles, which allows for increased energy production. Excess fission can also mark diseased mitochondria for mitophagy [2]. During fission, the mitochondria-fission 1 protein (Fis1) binds the GTPase dynamin-related protein 1 (DRP1) at the outer mitochondria membrane (OMM). GTPase activation of DRP1 triggers oligomerization and subsequently divides mitochondria [2]. Work has also shown that nitrosylation of DRP1 increases GTPase activity and causes mitochondrial fragmentation in human cell culture [3,4]. In contrast, mitochondria fusion involves the merging of a damaged mitochondria with another, healthier mitochondria, which allows for the complementation of damaged proteins by healthy ones, minimizing deleterious effects, such as ROS production [1]. Mitofusin (MFN)-1, -2 and optic atrophy 1 (OPA1) are GTPases that mediate OMM and inner mitochondria membrane (IMM) fusion, respectively [5]. GTPase activation of these proteins promotes fusion of the OMM, followed by fusion of the IMM [1]. In healthy cells, fission and fusion are constantly occurring and are in balance, while this delicate balance is disrupted during disease [3,4,5].

Work in mouse and human cell culture models of Huntington’s disease (HD) [1,6,7] showed altered levels of DRP1 and MFN2 [6], with the balance between fission and fusion tipped towards fission. Furthermore, pathogenic huntingtin (HTT) containing expanded polyQ repeats was bound more tightly to DRP1 than to normal HTT [8], with an increase in DRP1 GTPase activity [7,8]. Impaired calcium homeostasis [9] with increased cytoplasmic calcium and depletion of mitochondrial calcium stores [10] has also been reported in HD. Although mitochondria fragmentation is classically associated with oxidative stress [11,12], antioxidant treatments exacerbate neurodegeneration [12], while glutathione peroxidase activity has a protective effect in HD models [11]. Therefore, how mitochondria functions are disrupted in HD is still unclear. Here, we test the hypothesis that disruption of HTT function by excess pathogenic HTT impairs mitochondrial homeostasis in vivo. Using humanized *Drosophila* models of HD, polyQ disease or mechanically induced traumatic brain injury (TBI), together with pharmacological inhibitors, we show that disruption of HTT function is likely not required for mitochondrial defects, but that pathogenic polyQ alone is sufficient to cause DRP1-dependent mitochondrial fragments. Pathogenic polyQ-mediated mitochondrial defects are perhaps upstream of cell death, in contrast to mechanical stress/TBI-mediated mitochondrial fragmentation, which is likely caused by cell death. Taken together, our findings provide evidence for distinct physiological mechanisms for mitochondrial dysfunction in polyQ disease and mechanical stress/TBI.

## 2. Materials and Methods

### 2.1. Drosophila Genetics

Transgenic *Drosophila* lines UAS-HTT.15Q^C^-mRFP [13], UAS-HTT.138Q^C^-mRFP [13], UAS.HTT.FL.16Q/CyO (HTT.FL.16Q, BDSC, [14]), UAS-HTT.FL.128Q (HTT.FL.128Q, BDSC, [14]), UAS.HTTex1.25Q-eGFP [15], UAS.HTTex1.72Q-eGFP/CyO [15], HTTex1.103Q-eGFP [15], UAS-20Q [16], UAS-127Q [16], UAS-MJD.Q27 [17], UAS-MJD.Q78 [17], UAS-MJD.Q65-NLS/CyO [18], UAS-MJD.Q77-NES/CyO [18], UAS-HSPA (HSC70, BDSC) UAS-DRP1 (DRP1, BDSC), UAS-hMFN2/TM3;Sb1 (hMFN2, BDSC), DRP1KG03815/CyO (DRP1KG03815, BDSC), and UAS-PI3K.CAAX (PI3K.CAAX, BDSC) were used. Mitochondrial health reporters UAS-MitoTimer, UAS-Mito-roGFP2-ORP1 and UAS Mito-roGFP2-GRX1 (BDSC) were used. The pan-neuronal driver APPL-GAL4 was used for neuronal expression of all transgenic lines. Genetic crossings were done as in Gunawardena et al. [18]. Flies were raised at 29 °C and 60% humidity. In all cases, non-tubby female third-instar larvae were dissected. Sibling tubby larvae were evaluated as controls. Reciprocal crossings were also performed to confirm observations.

### 2.2. Larval Immunostaining and Quantification of Mitochondria Size

Third-instar larval brains were isolated via brain pull, in which larval segmental nerves remained attached. Larval brains were then fixed in 8% paraformaldehyde and immunostained (cytochrome C, 1:500, BD Biosciences or polyglutamine, 1:100, Fisher Scientific, Hampton, NH, USA; Appendix A). Larval nerves were imaged using a Nikon Eclipse TE 2000U microscope at 90× magnification (60× objective lens, 1.5× gain) (Nikon, Melville, NY, USA). A minimum of 250 individual axonal mitochondria were analyzed per genotype. At least six confocal optical images from the anterior, middle and posterior regions of six larvae were imaged, and the mitochondrial area was measured using NIH ImageJ as detailed in Krzystek et al. [19]. For axonal blockages, third-instar larvae were dissected as previously described in Gunawardena et al. [18] and then fixed in 8% paraformaldehyde and immunostained against cysteine string protein (CSP, 1:10, Developmental Studies Hybridoma Bank). Larval nerves were imaged under 40× magnification and the number of CSP-positive accumulations was determined using NIH ImageJ. At least six larvae were analyzed.

Co-localization was analyzed using Fiji (ImageJ, NIH) and Pearson’s coefficient (R) using the Coloc2 function from simultaneous images from HTT and CytC. R = 0 indicates no colocalization, while R = 1 indicates perfect colocalization. To identify the correlation between mitochondria size or number and HTT accumulation, R2 was determined via linear regression in Minitab. R2 = 0 indicates no correlation. −1 > R2 > 0 indicates a negative association, and 0 > R2 > 1 indicates a positive association.

### 2.3. TUNEL Assay

Third-instar larval brains were collected, fixed in 8% paraformaldehyde and permeabilized in 5% saponin prior to incubation with TdT enzyme/fluorescein-dUTP solution (In Situ Cell Death Detection Kit, Roche, Basel, Switzerland). Larval brains were imaged at 40× magnification. The number of TUNEL positive signals per area of brain was determined using NIH ImageJ, as detailed in Krzystek et al. [19]. A minimum of 5 brains were analyzed per genotype.

### 2.4. Mdivi-1 and L-NAME Treatment

For the Mdivi-1 incubation experiments, third-instar larval brains were dissected and incubated in buffer (0.01% DMSO) or 10 μM Mdivi-1 (Fisher Scientific) for 30 min prior to fixing with 8% paraformaldehyde and immunostaining. Larval brains were imaged and analyzed, as discussed previously [15]. For NO inhibition experiments, larvae were dissected in buffer (0.01% DMSO) or 0.5 M L-NAME (Fisher Scientific) prior to fixing and immunostaining, as in Kryzstek et al. [19].

### 2.5. In Vivo Mitochondria Health Reporters

Larvae expressing MitoTimer, Mito-roGFP2-ORP1, or Mito-roGFP2-GRX1 were dissected and imaged as previously described by Krzystek et al. [19]. MitoTimer-568 nm/MitoTimer-488 nm or Mito-roGFP2-488 nm/Mito-roGFP2-405 nm were simultaneously visualized using a NikonTE-2000E inverted fluorescence microscope with a beam splitter containing narrow single-band GFP/DsRED or YFP/GFP filters, a Cool Snap HQ cooled CCD camera, and a ProScan II high speed shutter (100 mm/s) for simultaneous imaging as detailed in Krzystek et al. [19]. For each larva, four sets of movies at an imaging window frame size of 100 µicrons at 150 frames were taken from the middle region of the larvae at an exposure of 500 ms. Kymographs were generated using Metamorph software, and 568/488 nm or 488/405 nm movies were split for analysis. From a total of 5 larvae, a set of 20 movies containing a cumulative total of >120 mitochondria were imaged for each genotype. Mitochondrial areas were measured as described above using NIH ImageJ. Relative 568/488 nm (colored: red/green) or 405 nm/488 nm (colored: ImageJ LUT-Fire, NIH) intensity ratios were obtained for each individual mitochondrial trajectory from each movie.

### 2.6. TMRM Assay

Third-instar larvae were dissected and treated with TMRM (200 nM, Thermo Fisher, Waltham, MA, USA) for one hour at room temperature [20]. The larval segmental nerves were imaged using a Nikon TE-2000E inverted fluorescence microscope equipped with a TxRed filter having excitation/emission wavelengths of 535/595 nm. The accumulation of TMRM dye in mitochondrial membranes depends on active mitochondria with intact membrane potential. Healthy, functional mitochondria provide a bright signal. Quantitative analysis of TMRM intensity was conducted using NIH ImageJ. A total of five larvae were imaged under each experimental condition.

### 2.7. JC-1 Assay

Third-instar larvae were dissected and treated with JC-1 (1:800, Cayman Chemicals, Ann Arbor, MI) for 10 min, as detailed in Krzystek et al. [19] and Wang et al. [21]. Subsequently, the larval segmental nerves were imaged using a Nikon TE-2000E inverted fluorescence microscope equipped with a beam splitter containing narrow single-band GFP/DsRED filters. The accumulation of JC-1 dye at mitochondrial membranes depends on the mitochondrial membrane potential (ΔΨm). Specifically, JC-1 exists in two distinct forms: as monomers (referred to as J-monomers) associated with low ΔΨm, with excitation/emission wavelengths of 485/535 nm, emitting green fluorescence, and as an aggregate form (referred to as J-aggregates) indicative of high ΔΨm, with excitation/emission wavelengths of 535/595 nm, emitting red fluorescence [22]. Quantitative analysis of red/green fluorescence intensity ratio was performed using NIH ImageJ to measure mitochondrial ΔΨm in larval segmental nerves. Five larvae were imaged and analyzed for each experimental condition.

### 2.8. Quantification of Nitric Oxide Levels

Third-instar larval brains were dissected and incubated in 10 µM 4-amino 5- methylamino-2′,7′-difluorescein diacetate (DAF-FM diacetate, Thermo Fisher, Waltham, MA, USA) for one hour at room temperature with agitation. As a control, the brains were incubated in PBS and used for background subtraction. DAF-FM diacetate-treated brains were washed in PBS and imaged with a Nikon TE-2000E inverted fluorescence microscope at 40× magnification. DAF-positive signals (fluorescent intensity relative to WT) were quantified using Fiji ImageJ. Five brains per genotype were analyzed.

### 2.9. Mechanical Stress-TBI by Vortexing

To induce mechanical shearing stress, individual larvae were placed in microcentrifuge tubes containing dissection buffer and vortexed at approximately 1800 rpm for five seconds prior to dissection. These larvae were immediately dissected and immunostained. Previously, in Krzystek et al. [19], we showed that vortexing caused mitochondrial fragmentation and damage in larvae expressing MitoTimer.

### 2.10. Western Blot and Analysis

Third instar larval brains were collected and homogenized in acetate buffer (10 mM HEPES, pH 7.4, 100 mM K acetate, 150 mM sucrose, 5 mM EGTA, 3 mM Mg acetate, 1 mM DTT, protease inhibitors (Pierce/Thermo Fisher, Waltham, MA), with phosphatase inhibitor (Pierce). The resulting homogenate was then centrifuged at 1000× *g* for 7 min at 4 °C. The resulting supernatant was then denatured (NuPAGE LDS), run on 10% Bis-Tris gels (Invitrogen/Thermo Fisher, Waltham, MA) and used for Western blotting (DRP1, 1:250 [23], MFN, 1:100 [24], or Tubulin, 1:1000, Abcam, Cambridge, UK). A minimum of 10–30 brains were collected per genotype. Images from 3 independent gels were analyzed using NIH ImageJ. 

### 2.11. Statistical Analysis

For immunofluorescence and TUNEL assays, all statistical analyses were performed in Minitab18 unless otherwise specified. First, power and sample size (n) calculations were performed for each experimental paradigm: comparing 2 means from 2 samples, with two-sided equality to identify the sample size that corresponds to a power of 0.9 with α = 0.05. At least 250 mitochondria were analyzed for each experiment. The appropriate statistical test was determined by evaluating the normality of the dataset using the Anderson–Darling test. For each experiment, significance was determined by performing the Kruskal–Wallis test, followed by Mann–Whitney pairwise comparisons, with α = 0.05. For western blots, all statistical analyses were performed in Microsoft Excel. Significance was determined using a two-sample two-tailed student’s *t*-test, α = 0.01.

## 3. Results

### 3.1. Excess Pathogenic Huntingtin Causes Mitochondrial Fragmentation in Drosophila Larval Axons

**Figure 1:**
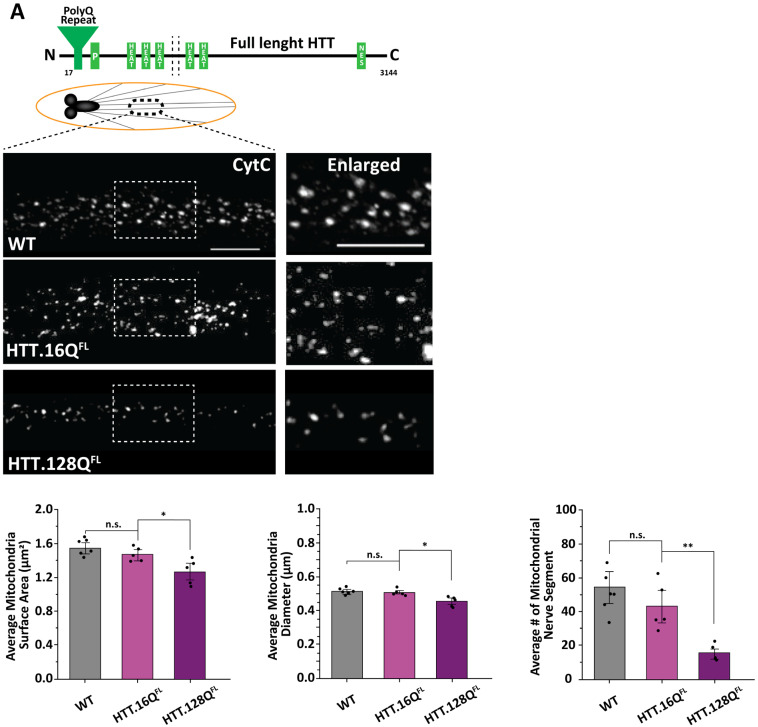

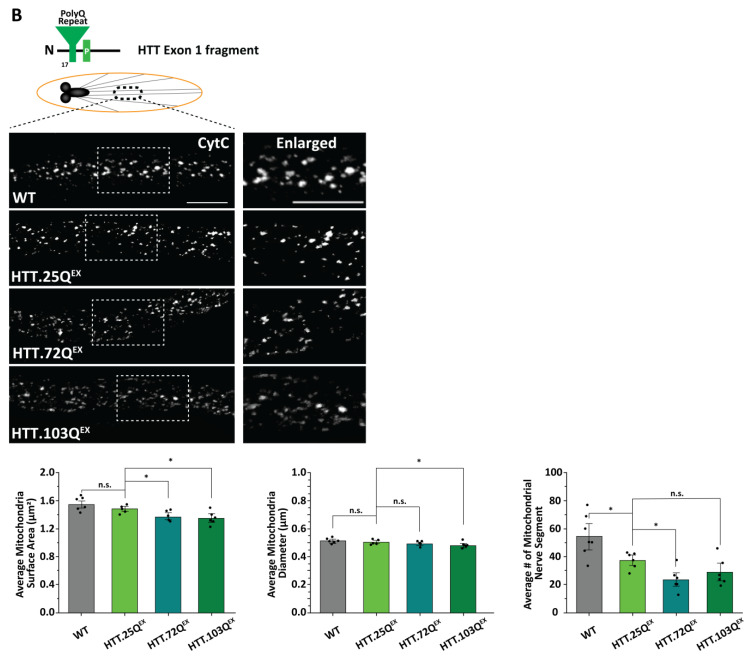

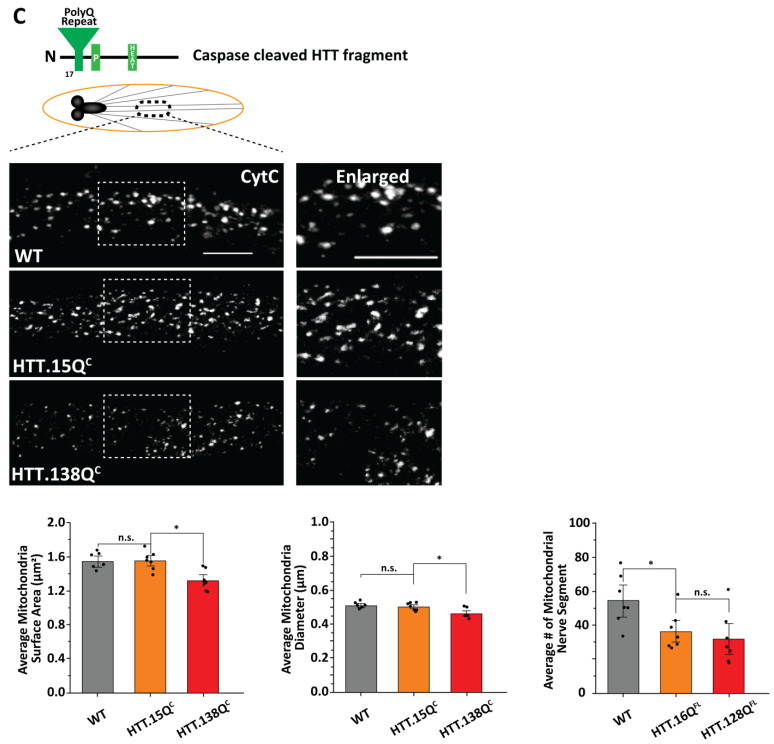
**Excess pathogenic HTT causes fragmented mitochondria.** (**A**) Schematic diagram of the full-length HTT protein (HTT^FL^). Representative images of larval nerves from the wild type (WT), larvae expressing non-pathogenic full-length HTT with 16 glutamine repeats (HTT.16Q^FL^), and larvae expressing full-length HTT with 128 glutamine repeats (pathogenic HTT.128Q^FL^) stained with CytC. Box shows enlarged area of the larval segmental nerve. Quantification analysis of mitochondrial surface area (µm^2^) (*p* = 0.037) and diameter (µm) (*p* = 0.012) in HTT.128Q^FL^ larvae compared to HTT.16Q^FL^ larvae. Quantification analysis of the number of mitochondria (*p* = 0.008) in HTT.128Q^FL^ larvae compared to HTT.16Q^FL^ larvae. Note that HTT.16Q^FL^ larvae show mitochondrial surface areas, diameters or numbers similar to WT. n = 5 larvae for each genotype. (**B**) Schematic diagram of the first exon of the HTT protein (HTT^ex1^). Representative images of larval segmental nerves from the wild type (WT), larvae expressing HTT^ex1^ containing 25 glutamine repeats (HTT.25Q^ex1^), HTT^ex1^ containing 72 glutamine repeats (HTT.72Q^ex1^) and HTT^ex1^ containing 103 glutamine repeats (HTT.103Q^ex1^) stained with CytC. Box shows enlarged area of the larval segmental nerve. Quantification analysis of mitochondrial surface area (µm^2^) (*p* = 0.031 HTT.72Q^ex1^, *p* = 0.020 HTT.103Q^ex1^), numbers (*p* = 0.018 HTT.25Q^ex1^, *p* = 0.02 HTT.72Q^ex1^) and diameters (µm) (*p* = 0.045 HTT.103Q ^ex1^) compared to HTT.25Q^ex1^ or WT larvae. n = 5 larvae for all genotypes. (**C**) Schematic diagram of the caspase-cleaved HTT fragment (HTT^C^). Representative images of larval nerves from wild-type (WT), larvae expressing caspase-cleaved HTT fragment with 15 glutamine repeats (HTT.15Q^C^), and larvae expressing pathogenic caspase-cleaved HTT fragment containing 138 glutamine repeats (HTT.138Q^C^) stained with CytC. Box shows the enlarged area of the larval segmental nerves. Quantification analysis of mitochondrial surface area (µm^2^) (*p* = 0.011) and diameter (µm) (*p* = 0.030) in HTT.138Q^C^ larvae compared to HTT.15Q^C^ larvae. Quantification of the number of mitochondria in HTT.15Q^C^ compared to WT (*p* = 0.021). n = 7 larvae for all genotypes. Scale bar = 5 μm. Statistical significance was determined by the Kruskal–Wallis test, followed by Mann–Whitney pairwise comparisons. * = *p* < 0.05, ** = *p* < 0.01, n.s. = *p* > 0.05.

To evaluate how pathogenic or expansion of poly-Q repeats in HTT causes mitochondria dysfunction in vivo, we expressed human full-length HTT with an expanded amount of polyQ repeats (pathogenic, HTT.Q128^FL^) in all neurons using the pan-neuronal GAL4 driver APPL-Gal4 and examined mitochondrial morphology using an antibody against cytochrome C, a protein loosely associated with the inner mitochondrial membrane in larval segmental nerves. Larvae expressing human full-length HTT with a normal amount of polyQ repeats (non-pathogenic, HTT.16Q^FL^) were used as a control. Adult flies expressing HTT.Q128^FL^ display phenotypes characteristic of HD, such as progressive neurodegeneration, motor impairment in the form of decreased climbing ability, and reduced lifespan [14,25]. Furthermore, HTT.128Q^FL^ expressing larval segmental nerves show axonal transport defects [26], pathogenic HTT accumulations and neuronal cell death in larval brains [26] (Figure S3). These larvae also showed fragmented mitochondria in contrast to HTT.16Q^FL^ and WT larvae (Figure 1A). Quantification analysis showed significant decreases in the mitochondrial surface areas (*p* = 0.037) and in the mitochondrial diameters (*p* = 0.012) in HTT.128Q^FL^ larvae compared to HTT.16Q^FL^ larvae (Figure 1A). Therefore, pathogenic full-length HTT can cause mitochondria fragmentation in vivo.

**Figure 2:**
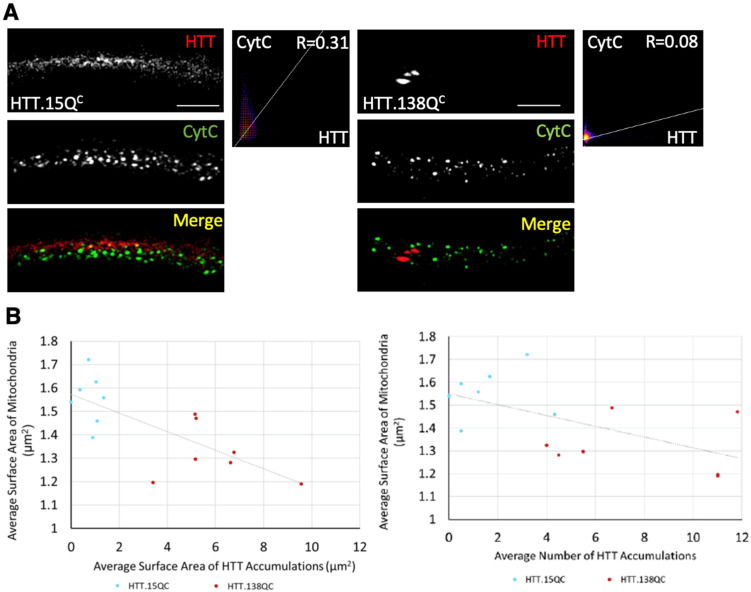
**HTT does not colocalize with mitochondria, but the extent of HTT accumulation correlates with mitochondrial fragmentation.** (**A**) Representative images of larvae expressing non-pathogenic normal HTT (HTT.15Q^C^) and pathogenic HTT (HTT.138Q^C^) in the context of mRFP (red) stained with CytC (green). The merged image has no yellow puncta. A co-localization pixel-heat map was generated from representative images as in A from 7 larvae. x-axis  =  HTT. y-axis  =  CytC. A line greater than 45 degrees with an R-value less than 1.0 corresponds to a low probability of co-localization. The R coefficient value between HTT and CytC for HTT.15Q^C^ was 0.31 and for HTT.138Q^C^ was 0.08. (**B**) Correlative comparison between the average surface area of mitochondria (µm^2^) and the average surface area of HTT accumulations (µm^2^) in HTT.15Q^C^ (blue) and HTT.138Q^C^ (red) show a negative correlation at R2 = 0.3451. Correlative comparison between the average surface area of mitochondria (µm^2^) and the average number of HTT accumulations in HTT.15Q^C^ (blue) and HTT.138Q^C^ (red) show a negative correlation at R2 = 0.5362. n = 7 larvae. Scale bar = 5 μm.

To evaluate whether the expression of the short pathogenic HTT^ex1^ fragment was sufficient to cause mitochondria fragmentation, we next examined larvae expressing pathogenic HTT^ex1^ (HTT.72Q^ex1^, HTT.103Q^ex1^) and larvae expressing non-pathogenic HTT^ex1^ (HTT.25Q^ex1^) in all neurons using the pan-neuronal GAL4, APPLGAL4. Expression of pathogenic HTT^ex1^ in *Drosophila* also showed HTT accumulation [15,27], decreased locomotion of adult flies, and decreased longevity [27]. Axonal transport defects and neuronal cell death were also seen in pathogenic HTT^ex1^ expressing larvae [18,26,28] (Figure 2). Larval segmental nerves from HTT.72Q^ex1^ or HTT.103Q^ex1^ expressing larvae show decreased mitochondria surface areas in contrast to larvae expressing non-pathogenic HTT^ex1^ (*p* = 0.031 and *p* = 0.020, respectively) (Figure 1B), indicating that the expression of pathogenic HTT^ex1^ is sufficient to cause mitochondria fragmentation.

Expression of pathogenic caspase-cleaved HTT (HTT.138Q^C^) caused HTT accumulations, decreased lifespan of adult flies, and decreased crawling ability in larvae compared to normal caspase-cleaved HTT (HTT.15Q^C^) [13]. HTT.138Q^C^ expressing larvae also contained axonal blockages in their segmental nerves and neuronal cell death in their brains [26,28] (Figure 2). Fragmented mitochondria with significant decreases in mitochondrial surface areas (*p* = 0.011) and diameters (*p* = 0.030) were also seen in HTT.138Q^C^ larval nerves compared to HTT.15Q^C^ larval nerves (Figure 1C). However, fragmentation does not appear to be the result of pathogenic HTT associated with mitochondria, since pathogenic HTT did not co-localized with mitochondria (Figure 2A). The extent of co-localization was quantified using Pearson’s R value. R = 1 indicates a high probability of co-localization compared to R = 0, which indicates no colocalization. The average Pearson’s R value for pathogenic HTT (HTT.138Q^C^) and mitochondria (CytC) was 0.08. Normal, wild-type (WT) HTT also did not colocalize with mitochondria (Figure 2A). The average Pearson’s R value for normal HTT (HTT.15Q^C^) and mitochondria (CytC) was 0.31. Therefore, expression of expanded polyQ repeats in the context of full length, exon 1 or a longer caspase cleaved fragment of HTT is sufficient to cause mitochondria fragmentation. Furthermore, while HTT is likely not normally present in mitochondria in vivo, mitochondrial fragmentation is not the result of defects in the normal function of HTT.

Previous work has shown that the length of polyQ repeats in HTT can directly correlate with the extent of axonal blockages in larval nerves [18], nuclear and cytoplasmic inclusions [18,19], the severity of disease phenotypes [19], and disease onset in humans [29]. Similarly, the extent of HTT accumulation also correlated with the extent of mitochondrial fragmentation (Figure 2B). Quantification analysis of the average number of HTT accumulations per larvae plotted against the average surface area of mitochondria per larvae showed a significant negative correlation (*p* = 0.027), indicating that increased numbers of HTT accumulations contribute to the extent of fragmented mitochondria. A similar trend was seen when comparing the average size of the HTT accumulations per larvae against the average surface area of mitochondria per larvae (*p* = 0.003, Figure 2B).

**Figure 3:**
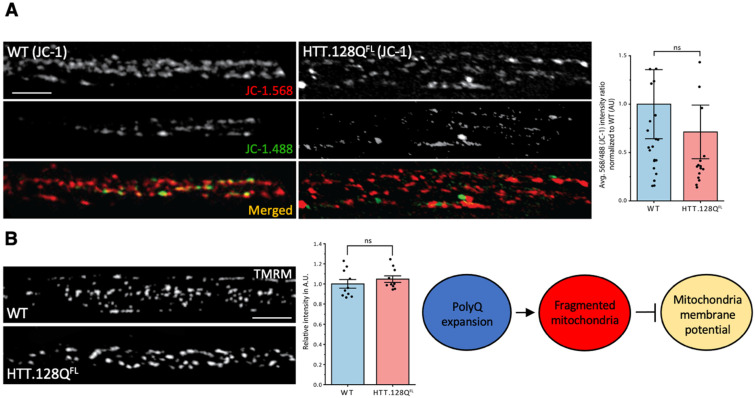
**Pathogenic HTT has no effect on mitochondrial membrane potential.** Representative segmental nerve images from wild-type (WT) larvae and larvae expressing HTT-128Q^FL^ stained with JC-1 (**A**), and TMRM (**B**). The green (488 nm) staining indicates JC-1 monomers, while red (568 nm) staining represents JC-1 aggregates. The red (568 nm)/green (488 nm) fluorescence signal ratio is used to determine healthy vs depolarized mitochondria. WT mitochondria are more red than green. The graph shows the quantification of the average 568 nm/488 nm intensity ratio in arbitrary units (A.U.) normalized to WT for HTT-128Q^FL^ mitochondria compared to WT mitochondria (*p* = 0.375). Healthier mitochondria show higher TMRM intensities, while damaged mitochondria show dimmer TMRM intensities. Both WT and HTT.128Q^FL^ show high intensities with TMRM. The graph shows the quantification of the relative intensity in arbitrary units (A.U.) normalized to WT for HTT-128Q^FL^ mitochondria (*p* = 0.389). n = 5 larvae per condition Scale bar = 5 µm. Statistical significance was determined using the Proc GLM procedure, followed by the pdiff mean comparison option in SAS Studio 3.81. n.s. = *p* > 0.05. The schematic diagram shows that pathogenic polyQ repeats can cause fragmented mitochondria but not defects in membrane potential.

While numerous studies have suggested that mitochondrial fragmentation is linked to mitochondrial dysfunction/oxidation [1,30], our previous work showed that this is not always the case since mitochondrial fragments induced by excess C-terminal truncated α-syn were not damaged or oxidized [19]. To test whether pathogenic polyQ HTT-mediated mitochondrial fragments were defective, we first measured mitochondrial depolarization using JC-1. WT control larvae showed a steady-state population of red and yellow JC-1 mitochondria (Figure 3A). Surprisingly, pathogenic polyQ HTT larvae (HTT.Q128^FL^) were similar to WT, with no significant differences seen in the average red (568 nm)/green(488 nm) intensity ratio normalized to WT (Figure 3A). Furthermore, mitochondrial depolarization using tetramethylrhodamine methyl ester (TMRM), a cell permeant dye that accumulates in active healthy mitochondria with intact membrane potentials, also failed to show defects in mitochondrial depolarization in HTT.Q128^FL^ (Figure 3B). Quantification of the relative intensity showed no significant changes between the WT and pathogenic polyQ HTT (Figure 3B). Therefore, pathogenic polyQ HTT-mediated mitochondrial fragments are likely not damaged.

### 3.2. Expansion of PolyQ Repeats Alone Can Cause Mitochondria Fragmentation

**Figure 4:**
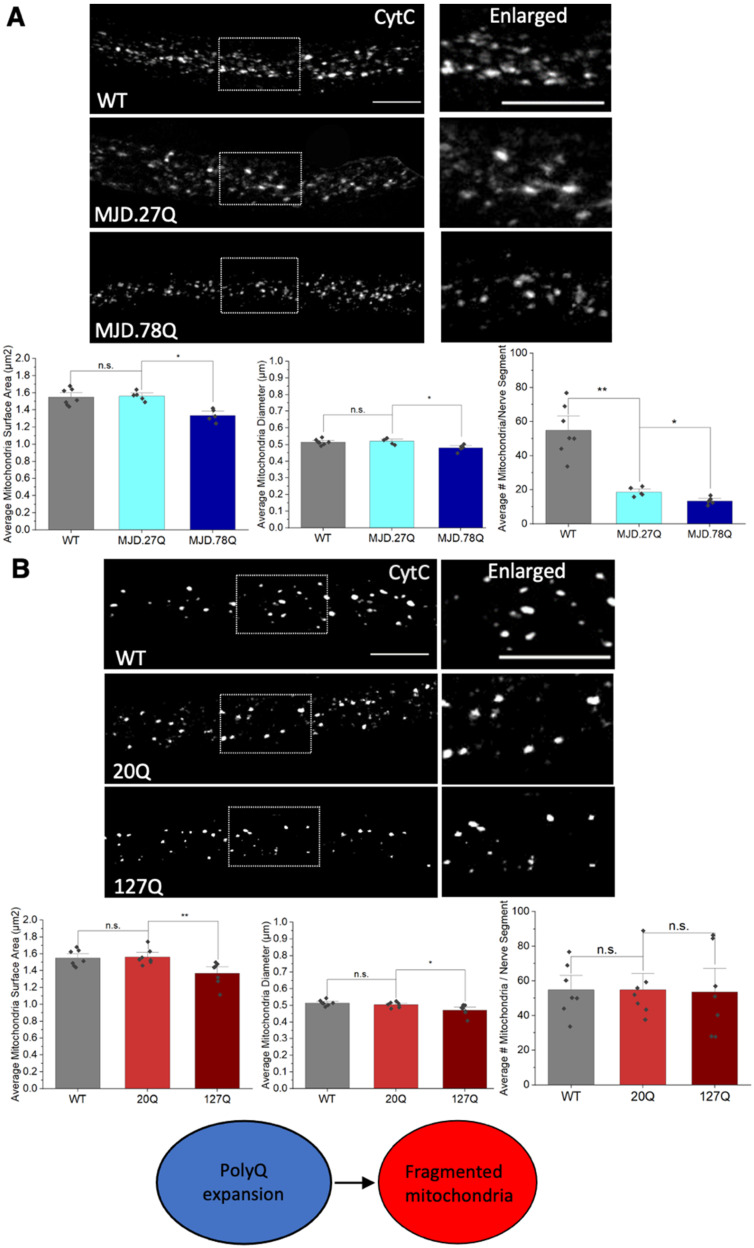
**Pathogenic polyQ repeats alone can cause mitochondrial fragmentation.** (**A**) Representative images from WT larvae and larvae expressing non-pathogenic (MJD.27Q) or pathogenic (MJD.78Q) polyQ repeats in the context of the MJD protein. Box shows enlarged area of the larval segmental nerve. Quantification of average mitochondrial surface areas (µm^2^) (*p* = 0.012), diameters (µm) (*p* = 0.022) or number of mitochondria (*p* = 0.006) in MJD.78Q larvae compared to MJD.27Q larvae. MJD.27Q larvae are comparable to WT. n = 5 larvae for each genotype. (**B**) Representative images of larvae expressing pathogenic polyQ repeats alone (127Q) or non-pathogenic polyQ repeats alone (20Q). Box shows enlarged area of the larval segmental nerve. Quantification analysis of the average mitochondrial surface areas (µm^2^) (*p* = 0.005) and diameters (µm) (*p* = 0.021) in 127Q larvae compared to 20Q larvae. Note that no significant changes were seen in mitochondrial numbers. n = 7 larvae for each genotype. Scale bar = 5 μm for all. Statistical significance was determined using the Kruskal–Wallis test, followed by Mann–Whitney pairwise comparisons. * = *p* < 0.05, ** = *p* < 0.01. n.s. = *p* > 0.05. The schematic diagram shows that pathogenic polyQ repeats alone can cause fragmented mitochondria.

To test the prediction that expansion of polyQ repeats alone can exert mitochondrial defects, we first examined larvae expressing expanded polyQ repeats in the context of MJD/SCA3, another neurodegenerative disease linked to the expansion of polyQ repeats. Flies expressing pathogenic polyQ repeats in the context of a truncated form of MJD (MJD.78Q) showed polyQ accumulations [18,31] (Figure S2), axonal transport defects [18], and neuronal death [18,31] (Figure S3). Larvae expressing MJD.78Q showed decreased mitochondria size (*p* = 0.012) and diameters (*p* = 0.022) compared to larvae expressing non-pathogenic MJD.27Q (Figure 4A). Since pathogenic polyQ expansion alone also caused axonal polyQ accumulations, axonal transport defects, and cell death [18,31] (Figure S3), we next evaluated larvae expressing 127Q repeats alone. Larvae expressing 127Q (pathogenic) repeats alone showed decreased mitochondrial surface areas (*p* = 0.005) and diameters (*p* = 0.021) compared to larvae expressing 20Q repeats (non-pathogenic polyQ) (Figure 4B). Furthermore, in contrast to our previous observations in larvae expressing α-syn [19], MJD.78Q-mediated fragmented mitochondria were not damaged or oxidized, as assayed by the in vivo mitochondrial turnover reporter MitoTimer and the in vivo mitochondrial oxidation reporters Mito-roGFP2-ORP1 and Mito-roGFP2-GRX1 (Figure S1). Therefore, pathogenic polyQ repeats alone are sufficient to induce mitochondrial fragmentation, but not damaged/oxidized mitochondria. Furthermore, fragmented mitochondria are not the result of defects in HTT or MJD protein functions.

### 3.3. PolyQ-Mediated Mitochondria Fragmentation Is Not Dependent on the Cellular Location of polyQ Accumulations

**Figure 5:**
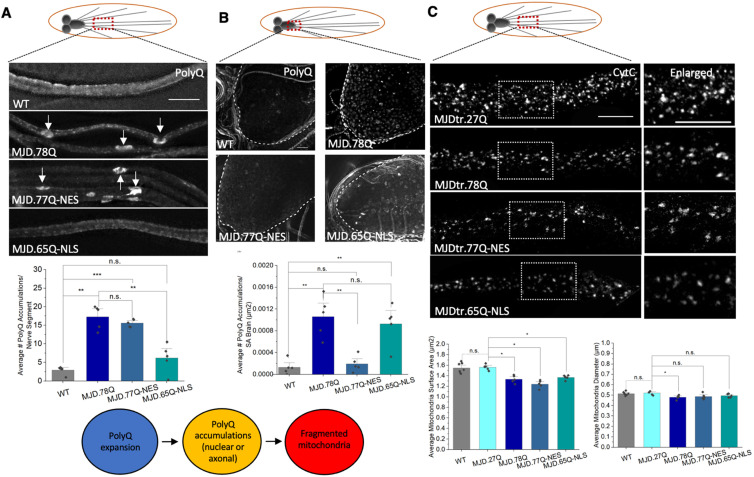
**Restricting pathogenic polyQ expression to the nucleus or the axonal cytoplasm does not influence mitochondrial fragmentation.** (**A**) Representative images from segmental nerves from larvae expressing pathogenic polyQ repeats in the context of MJD (MJD.78Q), pathogenic polyQ repeats in the context of MJD with a nuclear export signal (NES) (MJD.77QNES) or pathogenic polyQ repeats in the context of MJD with a nuclear localization signal (NLS) (MJD.65QNLS) stained with an antibody against polyQ. Note polyQ axonal blocks in MJD.78Q or MJD.77QNES larvae (arrows). MJD.65QNLS larvae have smooth staining similar to WT. Quantification of the average number of polyQ accumulations per nerve segment in MJD.78Q (*p* = 0.0002) and MJD.77QNES (*p* = 6.72 × 10^−8^) larvae compared to WT. n = 5 larvae for each genotype. Scale bar = 10 µm. (**B**) Representative images from MJD.78Q, MJD.77QNES or MJD.65QNLS larval brains stained with the polyQ antibody. Quantification analysis of the average number of polyQ accumulations per surface area (SA) of the brain (µm^2^) in MJD.78Q (*p* = 0.003) and MJD.65QNLS (*p* = 0.005) brains compared to WT. n = 5 larvae per genotype, scale bar = 10 µm. (**C**) Representative images from MJD.78Q, MJD.77QNES and MJD.65QNLS larval nerves stained with CytC. Box shows enlarged area of the larval segmental nerve. Quantification analysis of the average mitochondrial surface areas (µm^2^) in MJD.78Q (*p* = 0.012), MJD.77QNES (*p* = 0.012) or MJD.65QNLS compared to MJD.27Q. Note that the mitochondrial surface areas in MJD.27Q and WT are similar. Quantification analysis of the average mitochondrial diameters (µm) in MJD.78Q (*p* = 0.002) compared to MJD.27Q. n = 5 larvae for each genotype. Scale bar = 10 µm. Statistical significance was determined using the Kruskal–Wallis test, followed by Mann–Whitney pairwise comparisons. * = *p* < 0.05, ** = *p* < 0.01, *** = *p* < 0.001. n.s. = *p* > 0.05. The schematic diagram shows that pathogenic polyQ repeats can cause both nuclear and axonal polyQ accumulations, which can contribute to fragmented mitochondria.

To test whether the spatial localization of polyQ accumulations affects mitochondrial fragmentation, we examined larvae expressing pathogenic polyQ with either a nuclear localization sequence (MJD.65Q-NLS), which restricts polyQ to the nucleus or a nuclear export sequence (MJD.77Q-NES), which restricts polyQ to the cytoplasm. PolyQ accumulations were sequestered to the cell bodies in larvae expressing MJD.65Q-NLS, while axonal polyQ accumulations were seen in larvae expressing MJD.77Q-NES [18] (Figure 5A,B and Figure S2). Surprisingly, both larvae expressing MJD.65Q-NLS or MJD.77Q-NES showed mitochondrial fragmentation. Decreased axonal mitochondrial surface areas (*p* = 0.012) were seen in MJD.65Q-NLS larvae compared to MJD.27Q larvae, suggesting that sequestering polyQ to the nucleus does not prevent polyQ-mediated mitochondria fragmentation (Figure 5C). Decreased mitochondrial surface areas (*p* = 0.012) were also seen in MJD.77Q-NES larvae compared to MJD.27Q larvae (Figure 5C). Therefore, polyQ-mediated mitochondria fragmentation is not the result of the nuclear or axonal localization of polyQ accumulations.

### 3.4. Expression of HSP70 Chaperone Protein Rescues PolyQ-Mediated Mitochondrial Fragmentation

**Figure 6:**
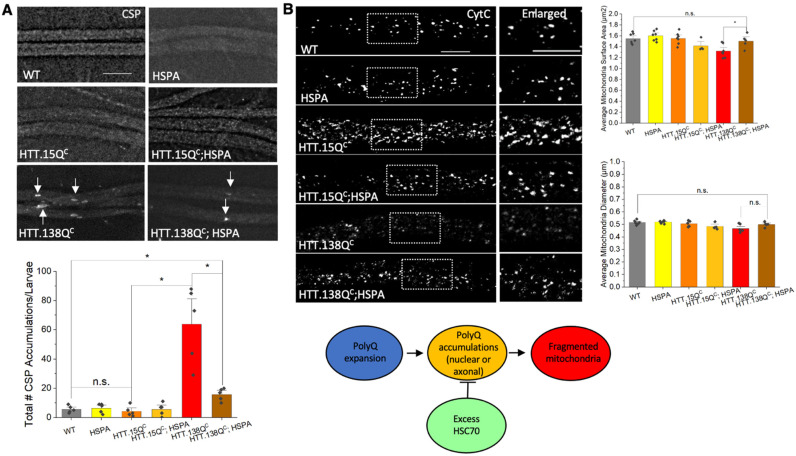
**PolyQ-mediated mitochondrial fragmentation is likely due to axonal accumulation.** (**A**) Representative images from larvae expressing HTT.138Q^C^, HTT.15Q^C^, the chaperone protein Hsc70 (HSPA) or WT stained with the synaptic vesicle protein CSP. Arrows show axonal accumulations/blockages. Few axonal accumulations/blocks are seen in HTT.138Q^C^ and HSPA co-expressing larvae. Quantification analysis of the total number of CSP accumulations per larvae in HTT.138Q^C^ (*p* = 0.012) larvae compared to HTT.15Q^C^ larvae and in HTT.138Q^C^ and HSPA co-expressing larvae compared to HTT.138Q^C^ (*p* = 0.011). n = 5 larvae per genotype. (**B**) Representative images of HSPA, HTT.15Q^C^, HTT.138Q^C^, and larvae co-expressing HSPA and HTT.15Q^C^ or HTT.138Q^C^ stained with CytC. Box shows the enlarged area of the larval segmental nerves. Quantification of the average mitochondrial surface area (µm^2^) in HTT.138Q^C^ and HSPA co-expressing larvae compared to HTT.138Q^C^ larvae (*p* = 0.035). n = 5 larvae per genotype. Scale bar = 5 μm. Statistical significance was determined using the Kruskal–Wallis test, followed by Mann–Whitney pairwise comparisons. * = *p* < 0.05. n.s. = *p* > 0.05. The schematic diagram shows that pathogenic polyQ repeats can lead to both nuclear and axonal polyQ accumulations, which can be modified by excess HSC70. Excess HSC70 can rescue polyQ-mediated fragmentation.

To test whether accumulation of misfolded toxic proteins results in mitochondria fragmentation, we expressed HSP70 (HSPA) in the context of pathogenic polyQ (HTT.138Q^C^). We previously showed that excess HSP70 rescued polyQ blockages within larval axons and neuronal cell death in larval brains [18], similar to what was seen by Warrick et al. [32] and in the context of α-syn aggregations [19,33] or SCA1 inclusions [34]. Similarly, larvae co-expressing HSP70 and HTT.138Q^C^ showed significant decreases in polyQ accumulations (*p* = 0.012) compared to HTT.138Q^C^ larvae (Figure 6A). Furthermore, mitochondrial surface areas were increased (*p* = 0.035) in larvae co-expressing HSP70 and HTT.138Q^C^ in contrast to larvae expressing HTT.138Q^C^ alone. Expression of HSP70 alone did not have any effect on mitochondrial size and these mitochondria were comparable to WT (Figure. 6B). Therefore, mitochondrial fragmentation is likely the result of misfolded pathogenic polyQ accumulations or aggregations.

### 3.5. PolyQ-Mediated Mitochondrial Fragmentation Is Not the Result of Cell Death

**Figure 7:**
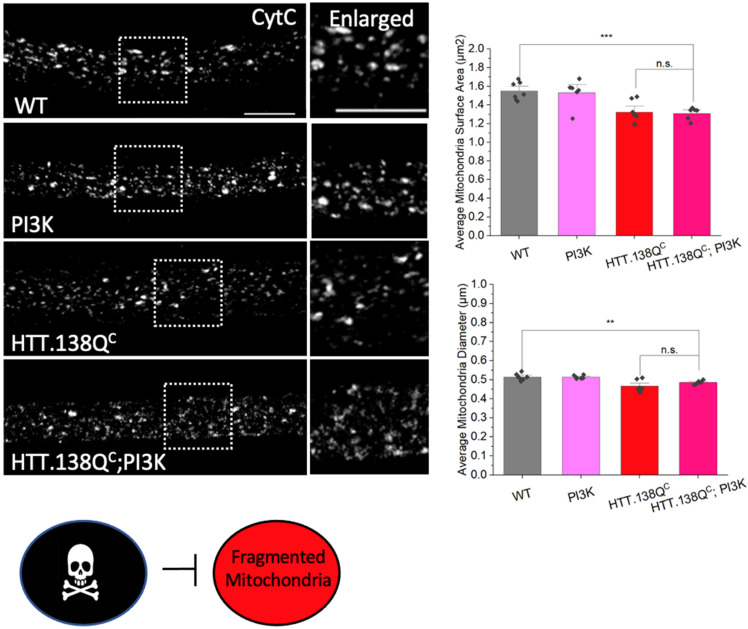
**PolyQ-mediated mitochondrial fragmentation is likely not the result of cell death.** Representative images from larvae expressing constitutively active PI3K (PI3K.CAAX), HTT.138Q^C^ or co-expressing HTT.138Q^C^ with PI3K.CAAX stained with CytC. Box shows enlarged area of the larval segmental nerve. Quantification analysis of the average mitochondrial surface area (µm^2^) and the average mitochondrial diameters in HTT.138Q^C^ and P13K.CAAX co-expressing larvae compared to WT larvae. n = 5 larvae per genotype. Scale bar = 5 μm. Statistical significance was determined using the Kruskal–Wallis test, followed by Mann–Whitney pairwise comparisons. ** = *p* < 0.01, *** = *p* < 0.001. n.s. = *p* > 0.05. The schematic diagram shows that cell death likely does not contribute to polyQ-dependent fragmented mitochondria. Excess P13K.CAAX does not affect polyQ-mediated fragmentation.

Cell death has been reported to trigger mitochondrial fragmentation [35]. Since activation of PI3K promoted a signaling cascade that increased protein synthesis and cellular proliferation [36], and because we previously showed that excess constitutively active PI3K (PI3K.CAAX) rescued cell death-mediated by pathogenic HTT [26], we rationalized that increasing components in the pro-survival PI3K/Akt pathway should also rescue pathogenic HTT-mediated mitochondrial fragmentation, if fragmentation is instigated by cell death. Surprisingly, we found that larvae expressing constitutively active PI3K (PI3K.CAAX) in the context of HTT.138Q^C^ still showed fragmented mitochondria (Figure 7). The average mitochondria surface areas and diameters in HTT.138Q^C^;PI3K.CAAX co-expressing larvae were similar to those seen in HTT.138Q^C^ expressing larvae (Figure 7). Therefore, pathogenic HTT-mediated mitochondrial fragmentation is likely independent of cell death.

### 3.6. PolyQ-Mediated Mitochondria Fragmentation Is Caused by an Imbalance of Fission and Fusion Proteins

**Figure 8:**
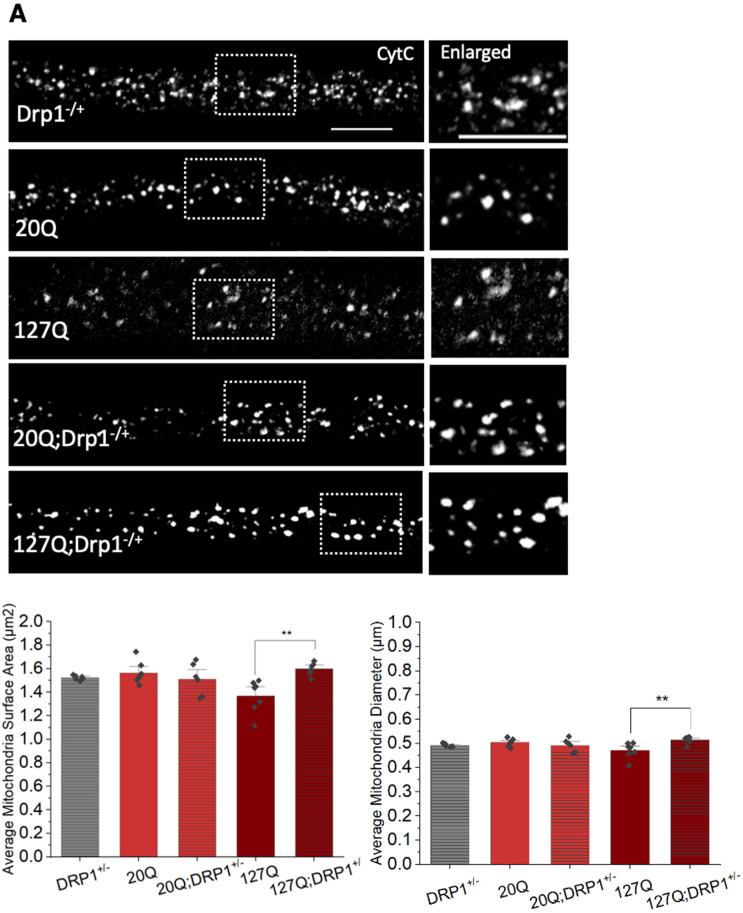

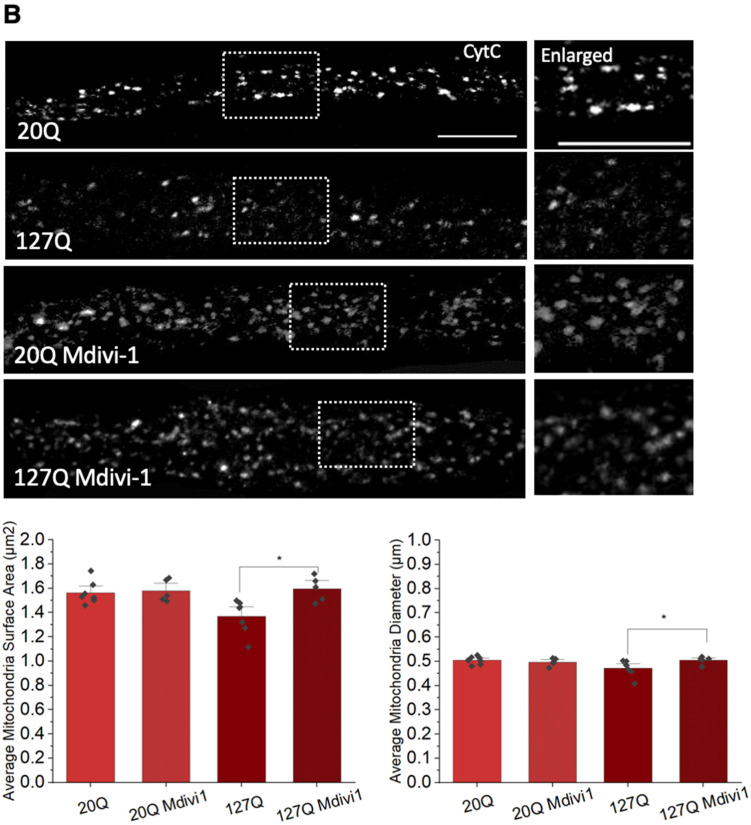

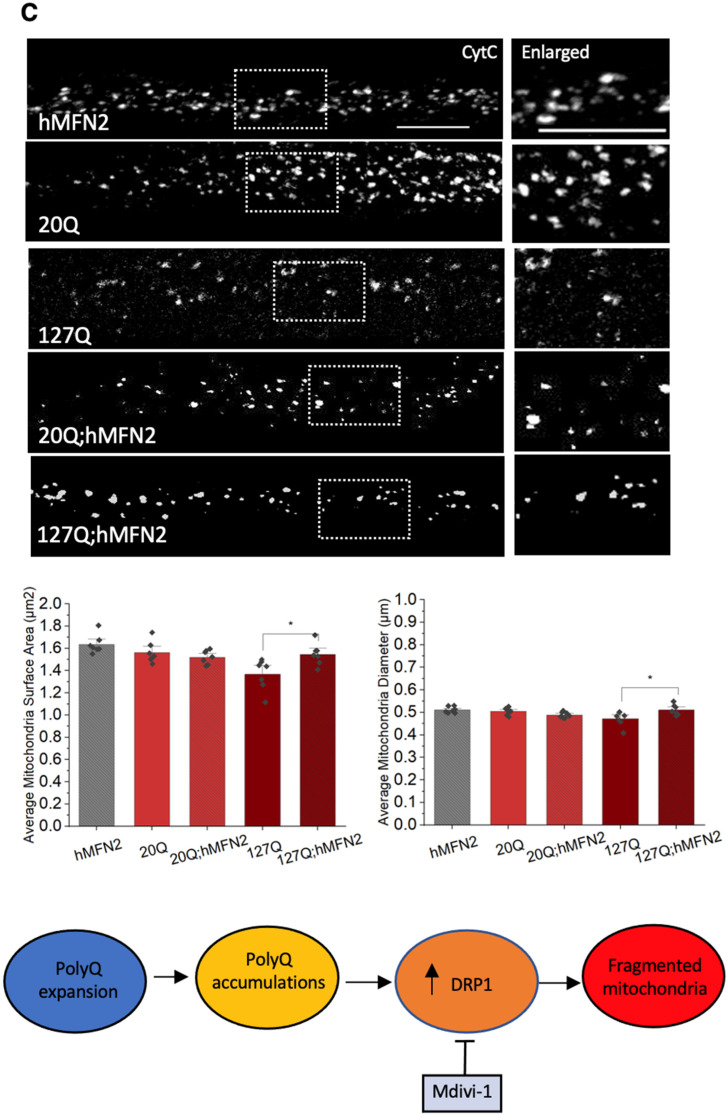
**PolyQ-mediated mitochondrial fragmentation is likely due to the imbalance of mitochondrial fission and fusion proteins.** (**A**) Represenatative images from heterozygous Drosophila DRP1 (Drp1^KG03815/+^ = Drp1−/+) mutant larvae, larvae expressing 20Q, larvae expressing 127Q and larvae 20Q (20Q; Drp1−/+) or 127Q (127Q;Drp1−/+) with the heterozygous DRP1 mutation stained with CytC. Box shows the enlarged area of the larval segmental nerves. Quantification analysis of the average mitochondrial surface area (µm^2^) in 127Q;Drp1−/+ larvae compared to 127Q larvae (*p* = 0.003). Quantification of the mitochondrial diameters in 127Q;Drp1−/+ larvae compared to 127Q larvae (*p* = 0.008). n = 6 larvae per genotype. (**B**) Representative images from larvae expressing 20Q or 127Q treated with either buffer or the DRP1 GTPase inhibitor Mdivi-1 at a 10 µM concentration and then stained with CytC. Box shows enlarged area of the larval segmental nerve. Quantification analysis of the average mitochondria surface areas (µm^2^) in 127Q Mdivi-1 treated larvae compared to 127Q buffer treated larvae (*p* = 0.015). Quantification of the average mitochondrial diameter (µm) in 127Q Mdivi-1 treated larvae compared to 127Q buffer treated larvae (*p* = 0.035). Note that the average mitochondria surface area or diameter in 20Q Mdivi-1 or 20Q buffer-treated larvae are similar. (**C**) Representative images from larvae expressing human MFN2 (hMFN2), 20Q and 127Q alone or larvae co-expressing hMFN with either 20Q (20Q;hMFN2) or 127Q (127Q;hMF2) stained with CytC. Box shows the enlarged area of the larval segmental nerves. Quantification analysis of the average mitochondria surface areas (µm^2^) in 127Q; hMF2 larvae (*p* = 0.021) compared to 127Q larvae. Quantification of the average mitochondrial diameters (µm) in 127Q;hMFN2 larvae compared to 127Q larvae (*p* = 0.03). Note that no changes are seen in the average mitochondria surface area or diameter in 20Q;hMFN2 larvae compared to 20Q larvae. n = 6 larvae per genotype. Scale bar = 5 μm. Statistical significance was determined using the Kruskal–Wallis test, followed by Mann–Whitney pairwise comparisons. * = *p* < 0.05, ** = *p* < 0.01. The schematic diagram shows pathogenic polyQ causes polyQ accumulations, which likely increase DRP1 levels to contribute to fragmented mitochondria. Mdivi-1 incubation rescues polyQ-mediated fragments.

A change in the level of fission and fusion proteins can either promote fission or inhibit fusion. Indeed, expression of DRP1 or increasing DRP1 GTPase activity caused mitochondria fragmentation in both Drosophila [4,19] and human cell culture [3], while expression of MFN caused mitochondria elongation in Drosophila [19]. Excess DRP1 alone caused mitochondria fragmentation, with decreased mitochondrial surface areas (*p* = 0.002) and diameters (*p* = 0.002) compared to WT [19] (Figure S2). Fission can also result due to the inhibition of MFN [37]. Indeed, loss of function of MFN in Drosophila caused small rounded mitochondria [37]. To test whether the polyQ-mediated mitochondrial fragmentation we observed was caused by changes to the balance of fission-fusion proteins, we expressed MFN in the context of pathogenic polyQ. Indeed, mitochondrial surface areas (*p* = 0.021) and diameters (*p* = 0.03) were increased in these larvae and mitochondria were now comparable to mitochondria in WT (Figure 8C). Furthermore, genetically decreasing the level of DRP using a loss of function mutant of DRP (DRP1^KG03815^) in the context of pathogenic polyQ also significantly increased mitochondria surface areas (*p* = 0.003) and diameters (*p* = 0.008) in contrast to fragmented mitochondria seen in larvae expressing pathogenic polyQ alone (Figure 8A). A similar effect was also seen when we treated larvae expressing pathogenic polyQ with the DRP1 GTPase inhibitor, Mdivi-1 (10 μM) (Figure 8B). Furthermore, biochemical analysis showed increased levels of DRP1, with no change in MFN levels in larval brains expressing pathogenic polyQ repeats compared to WT larval brains (Figure S4). Therefore, polyQ-mediated mitochondria fragmentation is likely due to an imbalance in the level of the fission protein DRP1. 

### 3.7. PolyQ-Mediated Mitochondria Fragmentation Is Likely Caused by Increased Levels of Nitric Oxide

**Figure 9:**
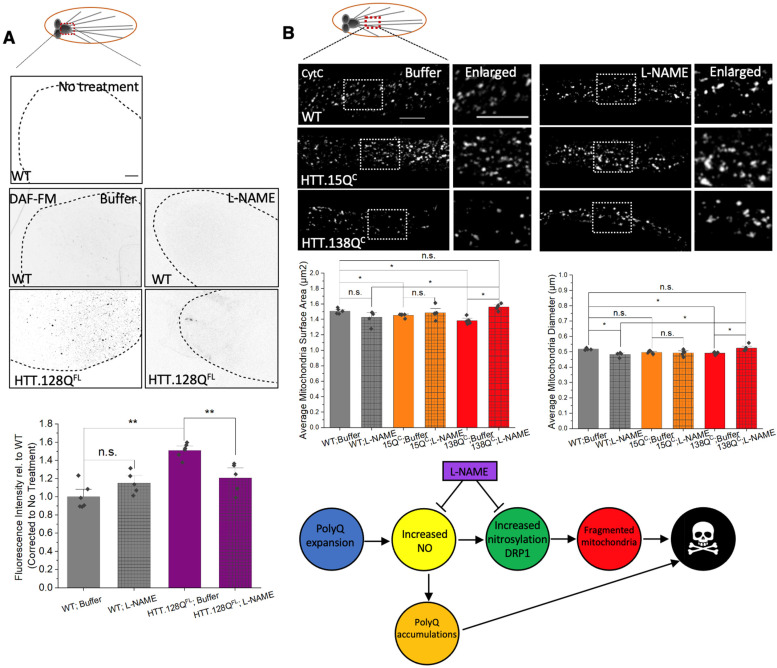
**PolyQ expansion increases nitric oxide (NO) levels. Pharmacological inhibition of NO production rescues polyQ-mediated mitochondrial fragmentation.** (**A**) Representative images of larval brains from WT and HTT.128Q^FL^ treated with DAF-FM after buffer or L-NAME incubation. No treatment represents no DAF-FM treatment to access background fluorescence levels. Quantitative analysis of the fluorescence intensity in buffer treated HTT.128^FL^ brains compared to WT buffer-treated brains (*p* = 0.005) and L-NAME treated HTT.128^FL^ brains compared to buffer treated HTT.128Q^FL^ brains (*p* = 0.008). (**B**) Representative images of WT, HTT.15Q^C^ and HTT.138Q^C^ larvae treated with either buffer or L-NAME stained with CytC. The box shows the enlarged area of the larval segmental nerve. Quantification analysis of mitochondrial surface areas (µm^2^) in HTT.138Q^C^ L-NAME treated larvae compared to buffer-treated larvae (*p* = 0.0122). L-NAME-treated WT or HTT.15Q^C^ larvae are comparable to buffer-treated WT or HTT.15Q^C^ larvae. Quantification analysis of the mitochondria diameters (µm) in L-NAME-treated compared to buffer treated HTT.138Q^C^ larvae (*p* = 0.0122). L-NAME-treated WT or HTT.15Q^C^ are comparable to buffer-treated WT or HTT.15Q^C^ larvae. n = 5 larvae per genotype. Statistical significance was determined using the Kruskal–Wallis test, followed by Mann–Whitney pairwise comparisons. * = *p* < 0.05, ** = *p* < 0.01. n.s. = *p* > 0.05. The schematic diagram shows pathogenic polyQ increases NO production, which may increase the nitrosylation of DRP1, which can result in polyQ-mediated fragmentation. Increases in NO can also increase polyQ accumulation. L-NAME incubation rescues polyQ-mediated fragments perhaps via decreasing NO production.

High concentrations of nitric oxide (NO) increased protein nitrosylation [38], and treatment with an NO donor caused mitochondria fragmentation in cell culture [3]. DRP1 is nitorsylated and nitrosylation of DRP1 can increase its GTPase activity, resulting in fragmented mitochondria [2,3,4]. Since high concentrations of NO have been reported in postmortem HD patient brains [4], perhaps mitochondrial fragmentation can occur via DRP1 nitrosylation due to increased NO [3,4]. Indeed, larvae expressing pathogenic HTT show significant levels of NO compared to WT larvae, as assayed by DAF-FM, which was quenched by L-NAME treatment, a selective nitric oxide synthase (NOS) inhibitor (Figure 9A). Intriguingly, L-NAME treatment rescued mitochondrial fragmentation in larvae expressing pathogenic HTT (Figure 9B). Increased mitochondrial surface areas (*p* = 0.012) and diameters (*p* = 0.012) were seen in L-NAME-treated pathogenic HTT larvae (HTT.138Q^C^) which were now similar to WT larvae (Figure 9B). In contrast, L-NAME treatment had no effect on mitochondrial morphology in larvae expressing non-pathogenic polyQ (HTT.15Q^C^) (Figure 9B) or in WT larvae. Therefore, increased levels of NO can contribute to polyQ-mediated mitochondrial fragmentation. 

### 3.8. PolyQ-Mediated Mitochondrial Fragments Are Likely Not the Result of Fragmentation Caused by Mechanical Stress

**Figure 10:**
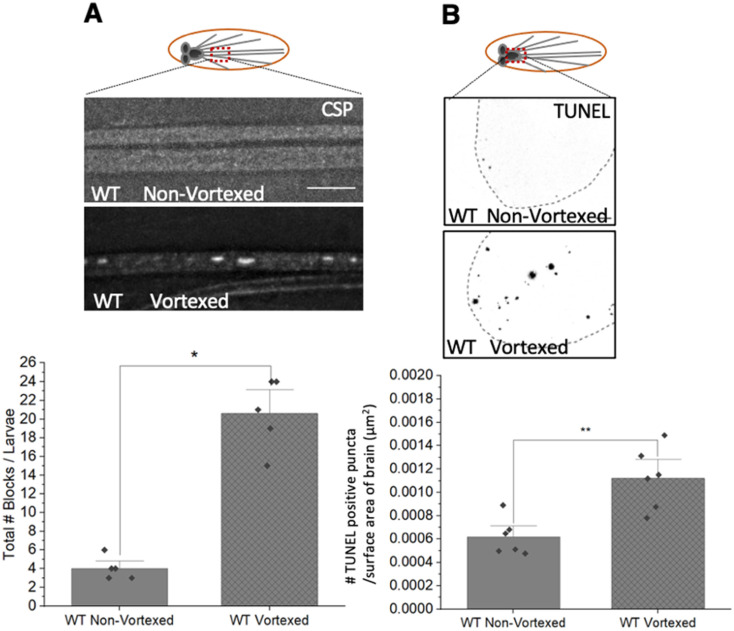

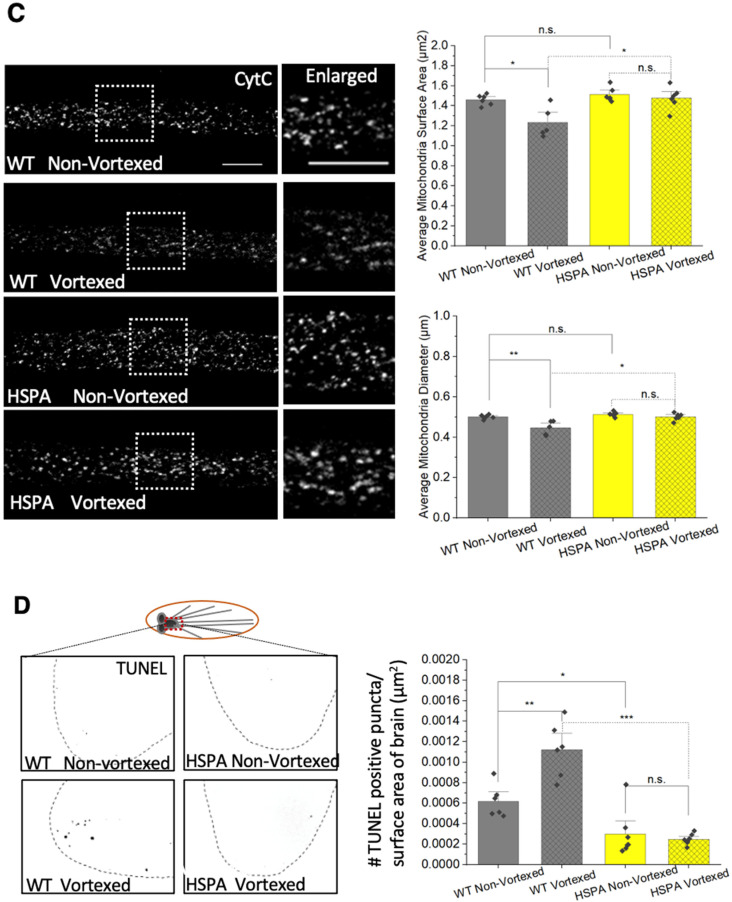

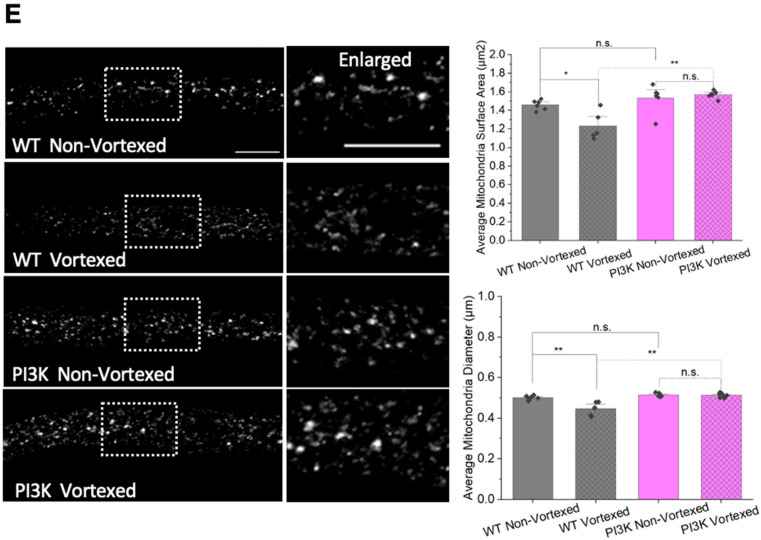

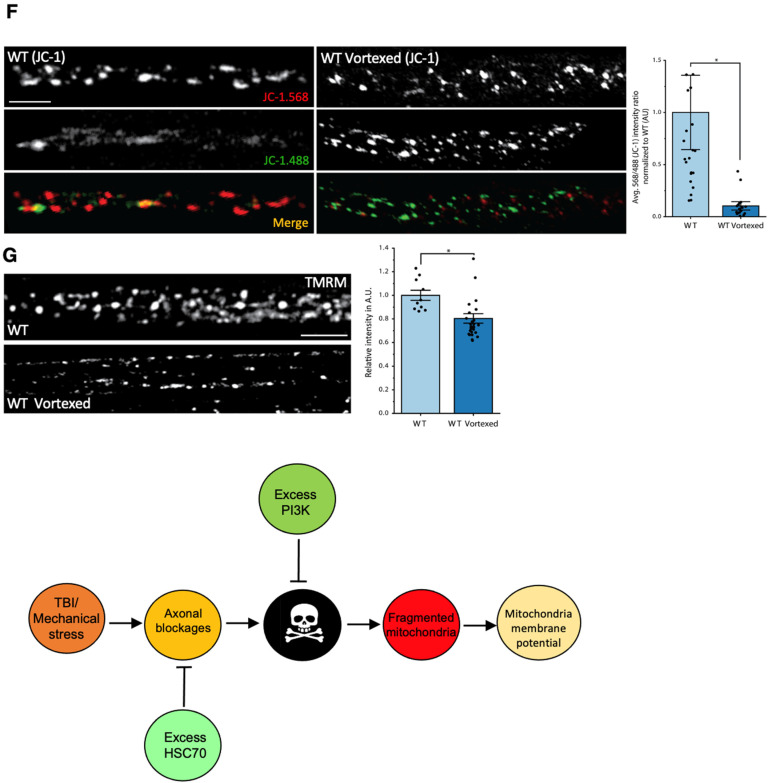
**Mechanical stress by vortexing causes axonal blockages, cell death, and damaged fragmented mitochondria. Expression of HSC70 but not P13KCAAX rescues mechanical stress-induced mitochondrial fragments.** (**A**) Representative images of vortexed and non-vortexed WT larvae stained with the synaptic antibody CSP to observe axonal blockages. Quantification of the total number of axonal blocks per larvae in vortexed larvae compared to non-vortexed larvae (*p* = 3.01 × 10^−6^). n = 5 larvae per condition. Scale bar = 10 μm (**B**) Representative images from larval brains from vortexed WT larvae and non-vortexed WT larvae stained with TUNEL reagents to access cell death. Quantification of the number of TUNEL-positive puncta per surface area (SA) of the brain (µm^2^) in vortexed larvae compared to non-vortexed larvae (*p* = 0.003). n = 5 larvae per condition. Scale bar = 10 μm (**C**) Representative images from vortexed WT and non-vortexed larvae and vortexed and non-vortexed HSC70 (HSPA) larvae stained with CytC. Box shows enlarged area of the larval segmental nerve. Quantification of the average mitochondrial surface area in vortexed larvae (*p* = 0.036) compared to non-vortexed larvae and vortexed HSC70 expressing larvae compared to vortexed WT larvae (*p* = 0.036). Quantification of the average mitochondrial diameters (µm) in vortexed larvae compared to non-vortexed larvae (*p* = 0.008) and vortexed larvae HSC70 larvae compared to vortexed WT larvae (*p* = 0.022). n = 5 larvae per condition. Scale bar = 5 μm (**D**) Representative images from vortexed and non-vortexed WT larval brains after the TUNEL assay. Quantification of the number of TUNEL-positive cells per surface area (SA) of the brain (µm^2^) from vortexed larvae compared to non-vortexed larvae (*p* = 0.01) and vortexed HSC70 larvae compared to vortexed WT larvae (*p* = 0.003). n = 5 larvae per condition. Scale bar = 10 μm (**E**) Representative images from vortexed and non-vortexed WT larvae and vortexed and non-vortexed PI3KCAAX larvae stained with CytC. Box shows enlarged area of the larval segmental nerve. Quantification analysis of average mitochondrial surface areas (µm^2^) from vortexed PI3KCAAX larvae compared to vortexed WT larvae (*p* = 0.006). Quantification of the average mitochondrial diameters (µm) from vortexed PI3KCAAX-expressing larvae compared to vortexed WT larvae (*p* = 0.006). n = 5 larvae per condition. Scale bar = 5 µm (**F**) Representative images from vortexed and non-vortexed WT larvae stained with JC-1. Quantification analysis of the average 568/488 nm JC-1 intensity ratio normalized to WT in arbitrary units (AU) in vortexed WT larvae compared to non-vortexed WT larvae (*p* = 0.002). n = 5 larvae per condition. Scale bar = 5 µm (**G**) Representative images from vortexed and non-vortexed WT larvae stained with TMRM. Quantification of the relative intensity of TMRM in arbitrary units (A.U.) from vortexed WT larvae compared to non-vortexed larvae (*p* = 0.01). n = 5 larvae per condition. Scale bar = 5 µm. The schematic diagram indicates that mechanical stress causes axonal blockages and cell death. Mechanical stress-induced cell death can cause mitochondrial fragmentation, which is rescued by excess PI3KCAAX. Mechanical stress-induced mitochondrial fragments are likely damaged. Statistical significance was determined using the Kruskal–Wallis test, followed by Mann–Whitney pairwise comparisons. * = *p* < 0.05, ** = *p* < 0.01, *** = *p* < 0.001. n.s. = *p* > 0.05.

Mitochondrial fragmentation has been linked to cellular stress [1]. Since polyQ accumulations can also cause cellular stress resulting in apoptosis [26], to test the proposal that polyQ-mediated mitochondrial fragmentation is directly the result of stress placed on the cell, perhaps by polyQ aggregations or inclusions, we examined mitochondrial morphology under a mechanical stress model of traumatic brain injury (TBI). Mechanical shearing caused by vortexing is a model of TBI in Drosophila [39]. Vortexed larvae showed decreased lifespan, synapse degradation, and cell death [39]. Significant amounts of axonal blockages (Figure 10A, *p* = 3.01 × 10^−6^) within vortexed larval segmental nerves and cell death (Figure 10B, *p* = 0.003) in vortexed larval brains were observed. Intriguingly, vortexed larvae also showed mitochondrial fragmentation, as assessed by significant decreases in mitochondria surface areas (*p* = 0.036) and diameters (*p* = 0.008) compared to non-vortexed larvae (Figure 10C). Strikingly, significant defects to the mitochondrial membrane potential were also seen with JC1-1 and TMRM (Figure 10F,G). Quantification of the average red (568 nm)/green(488 nm) intensity ratio of JC-1 normalized to WT showed a significantly deceased red/green ratio in vortexed larvae (Figure 10F, *p* = 0.002). Furthermore, quantification of the relative intensity of TMRM was also significantly decreased in vortexed larvae in contrast to WT larvae (Figure 10G, *p* = 0.01). These observations are similar to our previous work, which showed that vortexing larvae damaged mitochondria as assayed by the in vivo reporters MitoTimer, Mito-roGFP2-ORP1 and Mito-roGFP2-GRX1 [19]. Therefore, mechanical shearing by vortexing not only causes axonal transport defects and cell death but can also induce fragmented mitochondria that are damaged or unhealthy. 

While defects in axonal transport can trigger cell death, it has been proposed that cell death is downstream of axonal blockages [34]. Aberrant protein folding was implicated in axonal transport defects in many neurodegenerative diseases, including polyQ expansion diseases [18] and PD [19]. Since excess HSP70, which promotes proper protein folding, rescued polyQ-mediated axonal blockages, and cell death [18,19,34] (Figure 6A), we next examined larvae expressing HSP70 under mechanical stress. Intriguingly, larval brains expressing HSP70 failed to show cell death after vortexing, in contrast to WT larvae, which showed a significant amount of cell death after vortexing (*p* = 0.0003) (Figure 10D). Strikingly, mitochondrial fragmentation was also rescued in vortexed HSP70 expressing larvae with significantly increased mitochondrial surface areas (*p* = 0.036) and diameters (*p* = 0.022) (Figure 10C). Therefore, mechanical stress likely promotes protein misfolding, causing cell death and mitochondrial fragmentation. 

Our observations suggest that polyQ-mediated fragmentation is likely upstream of cell death since promoting pro-survival via the expression of PI3K.CAAX failed to rescue polyQ-mediated fragmentation (Figure 7), but rescued polyQ-mediated cell death [26]. However, several studies have shown that mitochondrial fragmentation can result due to cell death, as apoptotic signals can trigger excess fission, causing mitochondrial membrane depolarization and the release of cytochrome C from mitochondria [40,41]. To test the proposal that mechanical stress-induced cell death can cause mitochondrial fragmentation, we examined mitochondrial morphology in vortexed larvae expressing PI3K.CAAX. Significant rescue of mechanical stress-induced mitochondrial fragmentation was observed with increases in mitochondrial surface areas (*p* = 0.006) and diameters (*p* = 0.006) in vortexed PI3K.CAAX expressing larvae compared to vortexed WT larvae (Figure 10E). This is in contrast to what was seen in larvae co-expressing PI3K.CAAX and pathogenic polyQ, where pathogenic polyQ-mediated mitochondrial fragmentation was still present (Figure 7). Therefore, while fragmented damaged mitochondria can occur due to mechanical stress-mediated cell death, polyQ-mediated fragmented mitochondria that are not damaged are likely not the result of cell death. 

## 4. Discussion

Mitochondria dysfunction, including defective bioenergetic and morphological defects, is observed in postmortem HD brains and in several mouse models of HD [42,43]. However, how pathogenic HTT impairs mitochondrial homeostasis is unknown. Here, we identify that mitochondrial fragmentation is not restricted to HD but is likely the result of expanded polyQ repeats. Furthermore, polyQ-mediated mitochondrial fragments were not damaged/oxidized (Figure 1, Figure 3 and Figure 4) in contrast to mechanical stress (Figure 10) or excess α-syn induced mitochondrial fragments, which we previously reported [19] (Table S1). Moreover, polyQ-mediated fragmentation is not the result of cell death (Figure 7) but is likely a consequence of excess DRP1 (Figure 8), perhaps resulting from an increase in NO production (Figure 9). However, in contrast, cell death mediated by mechanical stress also contributes to mitochondrial fragmentation (Figure 10). Taken together, our observations reveal that distinct physiological mechanisms likely exist for how mitochondrial dysfunction occurs during polyQ-mediated degeneration and under mechanical stress conditions such as TBI.

### 4.1. PolyQ Expansion Is Sufficient to Induce Mitochondrial Fragmentation Independent of HTT and Is Likely Not the Result of Cell Death

Fragmentation of mitochondria was reported in lymphoblasts [6] and fibroblasts [7] derived from HD patients and with exogenous expression of pathogenic HTT in mice [7], *Drosophila* [43], and human cell culture [4]. Consistent with these studies, mitochondrial fragmentation was observed in larvae expressing full-length HTT, the first exon of HTT or the N-terminal fragment of caspase-cleaved HTT in the context of expansion of polyQ repeats (Figure 1). However, in contrast to what was previously reported, neither pathogenic HTT nor normal, wild type HTT was present with mitochondria in larval segmental nerves (Figure 2). While mutant HTT was observed in purified mitochondrial fractions, the majority of the wild-type or diseased/mutant HTT was seen in the cell homogenate and in the cytosolic fraction in human neuroblastoma cells and clonal striatal cells generated from HDQ111 homozygous knock-in mouse embryos [44]. Furthermore, sub fractionation under limited trypsin digestion of organelles indicated that mutant HTT can be associated with the outer membrane of mitochondria [44]. Another study postulated that specific N-terminal fragments of HTT can associate with mitochondria [45], however the N-terminus of HTT has a membrane association domain that facilitates interactions with different membranes [46,47]. Intriguingly, while low levels of full-length mutant HTT were seen in the mitochondrial fraction of young HD mouse brains, the amount of mutant HTT associated with mitochondria was observed to increase in older HD mouse brains [45], perhaps suggesting a progressive interaction with mitochondria that is dependent on increasing polyQ accumulations. While none of these studies looked at co-localization of mitochondria with HTT in neurons, Orr et al. [45] suggested that interactions between mutant HTT and mitochondria could interfere with the axonal distribution and rate of transport of mitochondria in cultured neurons. However, in contrast to this proposal, we failed to observe a decrease in the number of mitochondria located in axons with mutant HTT (Figure 1). Furthermore, we failed to detect either mutant or normal HTT co-localizing with mitochondria within larval axons (Figure 2), suggesting that perhaps direct associations between mutant or normal HTT and mitochondria could occur at other locations within neurons, in cell bodies or synapses, but not within axonal projections. Alternatively, mutant HTT could alter mitochondrial function indirectly by inhibiting the expression of transcriptional coactivators, which regulate mitochondrial biogenesis and respiration [48]. Regardless of this discrepancy, our observations suggest that expanded polyQ repeats are sufficient to induce mitochondrial fragmentation since fragmented mitochondria were observed in larvae expressing expanded polyQ repeats in the context of HTT (Figure 1), MJD or polyQ repeats alone (Figure 4). Furthermore, since the cellular localization of polyQ accumulations/aggregates did not directly correlate with the occurrence of fragmented mitochondria, as restricting polyQ accumulations to the nucleus using NLS or to the cytoplasm using NES failed to prevent mitochondrial fragmentation (Figure 5C), the location of polyQ accumulations is likely not important in the induction of mitochondrial fragments. Therefore, under physiological conditions, expanded polyQ repeats can instigate mitochondrial fragmentation via a common mechanism, and direct associations between pathogenic polyQ proteins and mitochondria are likely not required for mitochondrial defects seen in HD and other expanded polyQ repeat diseases. 

Previous work showed that fragmentation caused the accumulation of damaged mitochondrial components [1], increased ROS production [11,49,50,51,52,53], decreased cellular metabolism [51,52], resulting in cell death [18,26,51]. Increased oxidative stress was also seen in SCA2 patient fibroblasts [52]. SCA1 mouse models showed decreased oxidative activity and decreased cellular respiration [54,55]. Decreased metabolism and fragmented mitochondrial networks were reported in both SCA7 mouse models and patient fibroblasts [56]. Depolarized mitochondria was seen in SCA3/MJD mice, with increased mitochondria fragmentation, which correlated with decreased expression of fusion proteins MFN1/MFN2 and increased ROS production [57]. Since the expression of pathogenic polyQ was previously shown to cause decreased mitochondrial respiration and increased ROS production [51], perhaps polyQ-mediated mitochondrial fragmentation is a direct response to the stress placed on the cell by the presence of polyQ accumulations. Indeed, expression of a chaperone protein that rescued polyQ accumulations [18] significantly decreased polyQ-mediated mitochondrial fragments (Figure 6A). However, in contrast to what was observed with α-syn [19], polyQ-mediated mitochondrial fragments were not damaged or oxidized (Figure 3 and Figure S1). Furthermore, upregulation of the pro-survival pathway, which can prevent polyQ-mediated cell death [26] failed to rescue mitochondrial fragments (Figure 7). Therefore, while expanded polyQ repeats can cause cell death, mitochondrial fragmentation is likely not the result of dying neurons.

Mechanistically, mitochondrial fragmentation can result due to an imbalance in fission and fusion [1]. Indeed, increased levels of DRP1 were seen in pathogenic polyQ-expressing larval brains (Figure S5A). Both genetic reduction (loss of function, DRP1^KG03815^, Figure 8A) and pharmacological depletion of DRP1 (DRP1 GTPase inhibitor, Mdivi1, Figure 8B) rescued polyQ-mediated mitochondrial fragments, while excess DRP1 increased the amounts of fragmented mitochondria (Figure S4). These observations suggest that polyQ-mediated mitochondrial fragmentation is likely dependent on the overactivity of DRP1. On the other hand, decreased expression or activity of mitochondria fusion proteins, such as MFN1/2 or OPA1, could also induce fragmentation. Although decreased expression of MFN has been seen in both in vitro models of HD and MJD [6,57], we failed to see significant decreases in MFN levels (Figure S5B). However, an excess of MFN1 also rescued polyQ-mediated fragmentation (Figure 8C). Therefore, an imbalance in fission/fusion proteins likely contributes to the mitochondrial fragmentation seen in polyQ disease. 

Studies have shown that high levels of s-nitrosylated DRP1 can induce mitochondria fragmentation in rodents and cell culture [3,4], since nitrosylation of DRP1 increases DRP1 GTPase activity [3,4,7]. Furthermore, excess nitric oxide (NO) can result in increased nitrosylation of DRP1 [4]. Intriguingly, increased levels of NO together with elevated activity of DRP1 were reported in a number of stress conditions, including HD patients and mouse HD models [58], and TBI mouse models [59,60]. S-nitrosylation of DRP 1 has also been suggested to cause mutant huntingtin-induced mitochondrial fragmentation and neuronal injury in HD [4]. Consistent with these reports, we also observed increased NO in HTT-128^FL^ larval brains as assayed by DAF-FM, which was diminished by pharmacological inhibition of NO production using L-NAME (Figure 9B). Furthermore, L-NAME treatment rescued pathogenic polyQ-mediated mitochondrial fragmentation (Figure 9A). Therefore, excess NO likely contributes to polyQ-mediated mitochondrial defects. A similar proposal was suggested in AD with s-nitrosylation of DRP1 contributing to GTPase hyperactivity, resulting in mitochondrial fragmentation [3,4]. 

Alternatively, while increased amounts of NO can cause nitrosative stress, contributing to neuronal injury and cell death [61], depending on the disease process and the temporal sequence in which NO is involved, NO can also be neuroprotective by acting as an antioxidant during glucose deprivation and hypoxia [62]. Since polyQ-mediated mitochondrial fragmentation precedes neuronal cell death (Figure 6 and Figure 7), perhaps the increased NO and DRP1-dependent mitochondrial fragmentation we observe could be the result of an early response to neuroprotection. Indeed, in yeast, increasing mitochondrial biogenesis was sufficient to rescue polyQ-mediated cytotoxicity [63]. Indeed, the fact that we failed to observe polyQ-mediated effects on mitochondrial membrane potential (Figure 3) and the in vivo mitochondrial health markers neglected to show damaged or oxidized fragments (Figure S1) could perhaps hint at a scenario of increased mitochondrial biogenesis. Further study is needed to explore this possibility and to isolate the mechanisms of such a proposal.

### 4.2. PolyQ-Mediated Mitochondria Fragmentation Is Distinct from TBI-Mediated Fragmentation

Mitochondria are a major target of oxidative stress [23]. Stress conditions could arise due to pathogenic polyQ repeats, which are proposed to exert a gain of toxicity effect on cells. Formation of insoluble aggregates/accumulations which sequester other proteins into nuclear and cytoplasmic inclusions disrupts many cellular processes, including transcription, synaptic transmission, cellular trafficking, and signal transduction, as well as proteostasis and energy metabolism, culminating in neuronal cell death [18,26]. We previously demonstrated that expansion of polyQ repeats causes polyQ accumulations within axons that disrupt axonal transport [18] (Figure S2), instigate synaptic dysfunction [61] and neuronal cell death [26] (Figure S3). Expansion of polyQ repeats also causes fragmented mitochondria (Figure 1 and Figure 4), which is likely downstream of polyQ aggregates/accumulations, but upstream of cell death since expression of the HSP70 chaperon protein rescued mitochondrial fragments (Figure 6), but expression of anti-apoptotic, pro-survival proteins did not (Figure 7). Alternatively, since mitochondria can respond to and sense many stressors, mitochondrial fragmentation observed with pathogenic polyQ could be a result of cellular stress exerted by pathogenic polyQ. However, polyQ-mediated mitochondrial fragments were not damaged or oxidized (Figure 3 and Figure S1), in contrast to α-syn mediated fragments [19]. Similarly, fragmented mitochondria were also observed in a mechanical stress-induced TBI model (Figure 10), which can activate inflammatory and autophagy responses, increase Tau phosphorylation and induce neuronal defects disrupting sleep-related behaviors [39]. Strikingly, TBI-mechanical stress induced mitochondrial fragments were damaged/oxidized in contrast to polyQ-mediated fragments (Figure 10F,G). Axonal blockages and neuronal cell death were also observed in the TBI model (Figure 10). Expression of the chaperone protein HSP70 also rescued TBI/mechanical stress induced mitochondrial fragments (Figure 10C). However, in contrast to polyQ expansion, expression of pro survival proteins rescued TBI/mechanical stress induced mitochondrial fragments (Figure 10E). Therefore, unlike polyQ expression, TBI/mechanical stress induced mitochondrial fragmentation is likely a consequence of cell death. 

In conclusion, our observations suggest that mitochondria can become fragmented due to divergent mechanisms. One limitation of our study is isolating how aging contributes to polyQ-mediated mitochondrial fragmentation since it is possible that polyQ-mediated mitochondrial fragments could progressively become damaged over time. Further study is needed to isolate the individual mechanistic details of how mitochondria become fragmented under different cellular stress conditions or diseases and how aging contributes to these processes. 

## Data Availability

All data generated or analyzed during this study are included in this published article or in supplementary information. Raw data is available from the corresponding author upon request.

## Supplementary Materials

The following supporting information can be downloaded at: https://www.mdpi.com/article/10.3390/cells12192406/s1, Figure S1: Expression of pathogenic polyQ repeats in the context of MJD causes mitochondria fragmentation; Figure S2: Expression of pathogenic polyQ repeats cause axonal blockages; Figure S3: Expression of polyQ repeats cause cell death.; Figure S4: Excess fission protein DRP1 has no effect on polyQ-mediated mitochondrial fragmentation; Figure S5; Western blot analysis of DRP1 and MFN in pathogenic polyQ-expressing larval brains. Table S1: Comparative summary of HD/polyQ and TBI/mechanical stress conditions.

## Appendix A. Key Resources Table

**Resource**	**Source**	**Identifier**
***Antibodies and Dyes***		
**Mouse anti-Cytochrome C (7H8.2C12)**	BD Pharmingen	Cat#556433 RRID: AB_396417
**Mouse anti-Polyglutamine**	Fisher Scientific	Cat# MAB1574MI RRID: N/A
**Rabbit anti-DRP1**	Laboratory of Leo Pallanck	Poole et al., 2010 [64]
**Rabbit anti-Marf**	Laboratory of Alex Whitworth	Zivani et al., 2010 [24]
**Mouse anti-Tubulin (DM1A)**	Abcam	Cat#ab7291 RRID: AB_2241126
**Anti-Mouse Alexa Fluor^®^ 488**	Thermo Fisher	Cat# A1101 RRID: AB_2534069
**Anti-Mouse Alexa Fluor^®^ 568**	Thermo Fisher	Cat# A1104 RRID: AB_2534072
**Anti-Mouse secondary antibody, HRP**	Thermo Fisher	Cat# 32430 RRID: AB_1185566
**Anti-rabbit secondary antibody, HRP**	Thermo Fisher	Cat# 32460 RRID: AB_1185567
**In situ Cell Death Detection Kit, Fluorescein**	Roche	Cat#11684795910 Version #17
**DAF-FM Diacetate**	Thermo Fisher	Cat# D23844 RRID: N/A
**TMRM**	Thermo Fisher	Cat# I34361 RRID: N/A
**JC-1**	Cayman Chemical	Cat# 10009172 RRID: N/A
***Chemicals, Peptides, and recombinant proteins***		
**Protease inhibitor cocktail**	Pierce	Cat# PIA32965 RRID: N/A
**Phosphatase Inhibitor**	Pierce	Cat# P188667 RRID: N/A
**VectaShield Mounting Medium**	Fisher	Cat# NC9265087 RRID: N/A
**Mitochondrial Division Inhibitor, Mdivi-1**	Fisher Scientific	Cat# 47-585-610MG PubChem: 33825829
**L-NAME hydrochloride**	Fisher Scientific	Cat# 06-651-00 PubChem: 135193
***Experimental Models: D. melanogaster organisms/strains***		
**P{w[+m*]=Appl-GAL4.G1a}1, y [1] w[*]**	Bloomington Drosophila Stock Center	BDSC: 32040 FlyBase: FBst0032040
**Appl-GAL4; T(2,3), CyO, TM6B, Tb1/Pin88k**	Laboratory of Lawrence Goldstein	Gunawardena and Goldstein, 2001 [34]
**UAS-HTT.15Q.mRFP**	Laboratory of J Troy Littleton	Weiss et al., 2012 [13]
**UAS-HTT.138Q.mRFP**	Laboratory of J Troy Littleton	Weiss et al., 2012 [13]
**w[1118]; P{w[+mC]=UAS-HTT.16Q.FL}F24/CyO**	Bloomington Drosophila Stock Center	BDSC: 33810 FlyBase: FBst0033810
**w[1118]; P{w[+mC]=UAS-HTT.128Q.FL}f27b**	Bloomington Drosophila Stock Center	BDSC: 33808 FlyBase: FBst0033808
**UAS-HTT.25Q-eGFP**	Laboratory of Dr. Norbert Perrimon	Zhang et al., 2010 [15]
**UAS-HTT.72Q-eGFP**	Laboratory of Dr. Norbert Perrimon	Zhang et al., 2010 [15]
**UAS-HTT.103Q-eGFP**	Laboratory of Dr. Norbert Perrimon	Zhang et al., 2010 [15]
**UAS-20Q**	Laboratory of Seymour Benzer	Kazemi-Esfarjani and Benzer, 2002 [16]
**UAS-127Q**	Laboratory of Seymour Benzer	Kazemi-Esfarjani and Benzer, 2002 [16]
**UAS-MJD.27Q**	Laboratory of Nancy Bonini	Bonini, 1999 [17]
**UAS-MJD.78Q**	Laboratory of Nancy Bonini	Bonini, 1999 [17]
**UAS-MJD.Q77-NES/Cyo**	Laboratory of Nancy Bonini	Gunawardena et al., 2003 [18]
**UAS-MJD.Q65-NLS/Cyo**	Laboratory of Nancy Bonini	Gunawardena et al., 2003 [18]
**w^1118^; P{UAS-Hsap\HSPA1L.W} 41.1**	Bloomington Drosophila Stock Center	BDSC: 7454 FlyBase: FBst0007454
**y^1^; P{SUPor-P} Drp1^KG03815^/CyO; ry^506^**	Bloomington Drosophila Stock Center	BDSC: 13510 FlyBase: FBst0013510
**w^1118^; P{UAS-hMFN2.D}29/TM3, Sb^1^**	Bloomington Drosophila Stock Center	BDSC: 59044 FlyBase: FBst0059044
**P{w[+mC]=UAS-Pi3K92E.CAAX}1, y[1] w[1118]**	Bloomington Drosophila Stock Center	BDSC: 8294 FlyBase: FBst0008294
**w^*^; P{UAS-Drp1.D}3**	Bloomington Drosophila Stock Center	BDSC: 51647 FlyBase: FBst0051647
**UAS-Mitotimer**	Bloomington Drosophila Stock Center	BDSC: 57323 FlyBase: FBst0057323
**UAS-Mito-roGFP2-Grx1**	Bloomington Drosophila Stock Center	BDSC: 67664 FlyBase: FBst0067664
**UAS-Mito-roGFP2-ORP1**	Bloomington Drosophila Stock Center	BDSC: 67667 FlyBase: FBst0067667
***Software/algorithms***		
**ImageJ**	Schneider et al., 2012 [65] https://imagej.net/. Accessed on 2 October 2023.	RRID: SCR_003070
**Metamorph/Metavue Imaging Software**	Molecular Devices, Sunnyvale, CA, USA	RRID: SCR_002368
**Minitab 18**	https://www.minitab.com/en-us/. Accessed on 2 October 2023.	RRID: SCR_014483
**Microsoft Excel**	https://www.microsoft.com/en-gb/. Accessed on 2 October 2023.	RRID: SCR_016137
**OriginLab/OriginPro**	https://www.originlab.com/. Accessed on 2 October 2023.	RRID: SCR_014212
