# Supplementary material for: PolyQ-Expansion Causes Mitochondria Fragmentation Independent of Huntingtin and Is Distinct from Traumatic Brain Injury (TBI)/Mechanical Stress-Mediated Fragmentation Which Results from Cell Death
Source: Cells. 2023 Oct 5;12(19):2406. doi: 10.3390/cells12192406 (PMC10572422; doi:10.3390/cells12192406)
Supplement: Supplementary file 1 [file cells-12-02406-s001.zip › cells-2575794-supplementary.pdf]

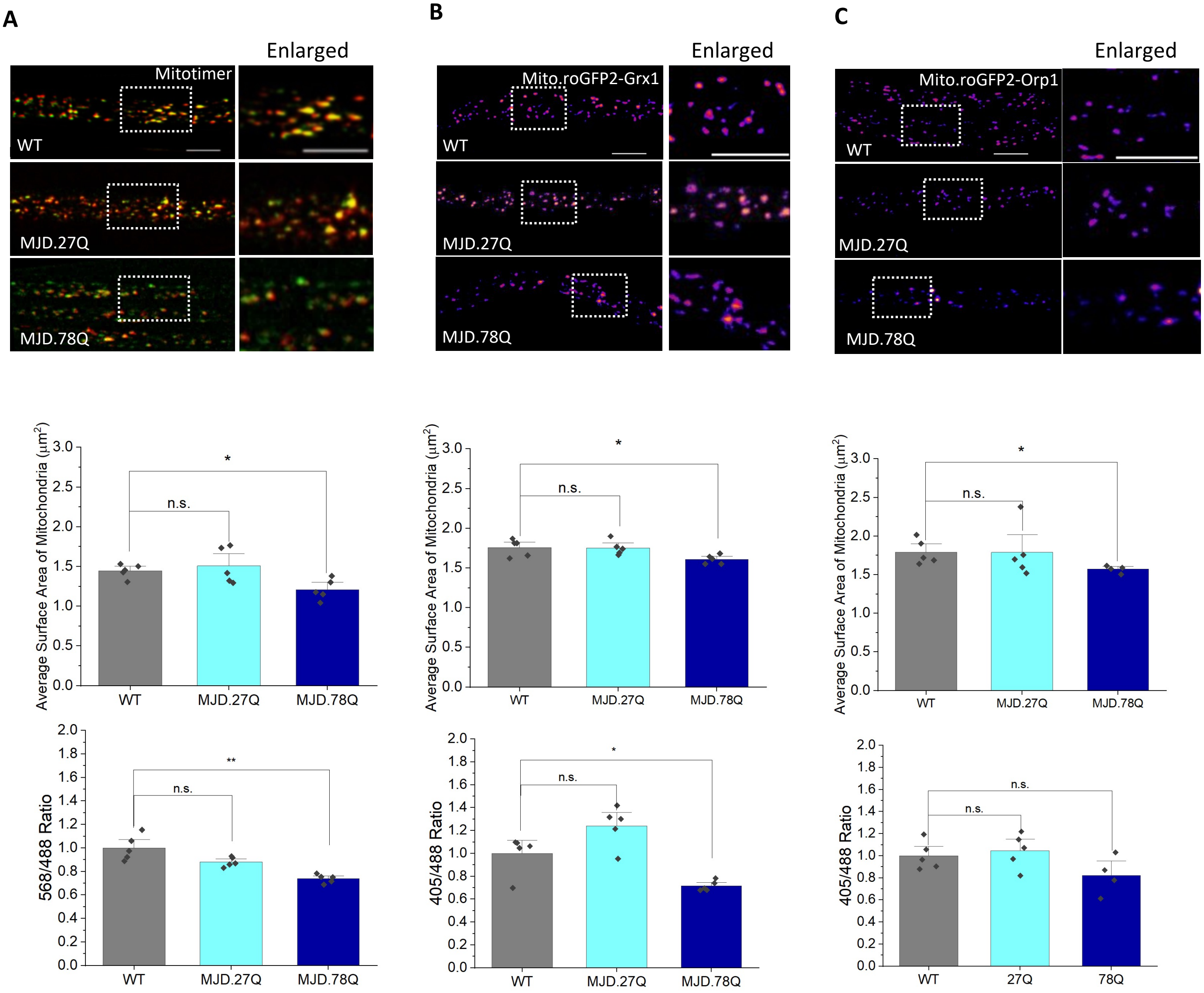

Figure S1. Pathogenic polyQ repeats in the context of MJD causes mitochondria fragmentation. Representative images from larvae co-expressing pathogenic polyQ repeats in the context of MJD (MJD.78Q) and mitochondrial health markers MitoTimer which report damaged mitochondria in red and healthy mitochondria in green, MitoroGFP2-Grx1 the glutathione redox sensor or MitoroGFP2-Orp1 the H2O2 redox sensor. Box shows enlarged area of the larval segmental nerves. Quantification analysis of average mitochondrial surface areas (μm^2) from larvae expressing MJD.78Q with MitoTimer (p=0.013) (**A**), or MitoroGFP2-Grx1 (p=0.036) (**B**) or MitoroGFP2-Orp1 (p=0.032) (**C**). Quantification of the 568/488 ratio in MJD.78Q;MitoTimer larvae compared to MitoTimer (WT) larvae (p=0.003) (**A**), the 405/488 ratio in MJD.78Q;MitoroGFP2-Grx1 compared to MitoroGFP2-Grx1 (WT) larvae (p=0.017) (**B**) or the 405/488 ratio in MJD.78Q;MitoroGFP2-Orp1 compared to MitoroGFP2-Orp1 (WT) larvae (**C**). n=5 larvae per genotype. Scale bar=5μm for all. Statistical significance was determined using Krushkall-Wallis test followed by Mann-Whitney pairwise comparisons. *=p<0.05, n.s.=p>.05

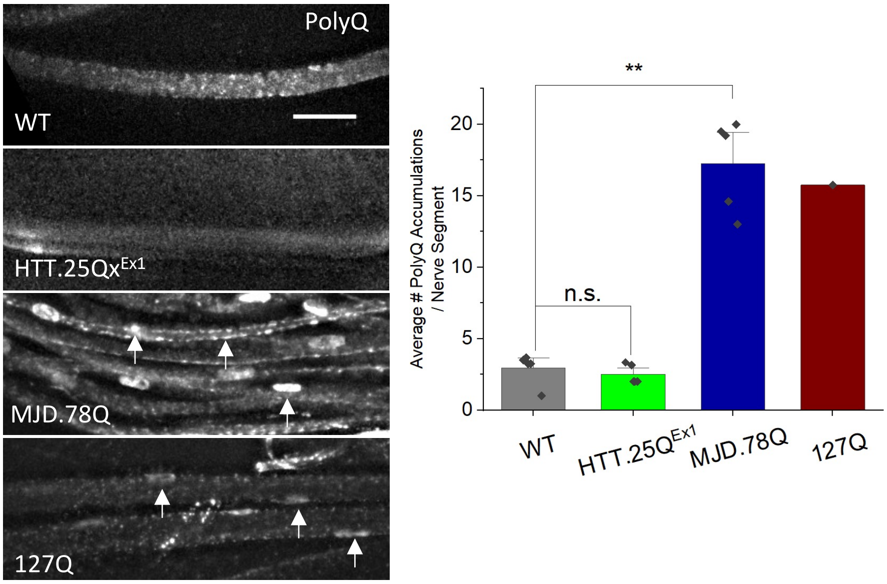

Figure S2. Pathogenic polyQ repeats cause axonal blockages. Representative images from larvae expressing MJD.78Q, 127Q and HTT.25Q^{ex1} stained with an antibody against polyQ. Quantitative analysis of the average number of polyQ accumulations (arrows) per nerve segment in MJD.78Q larvae ($p=0.012$, $n=5$ larvae) and 127Q larvae compared to WT ($n=1$ larvae). Scale bar=10µm. Statistical significance was determined via Kruskall-Wallis test followed by Mann-Whitney pairwise comparisons. **=$p<0.01$, n.s.=$p>0.05$

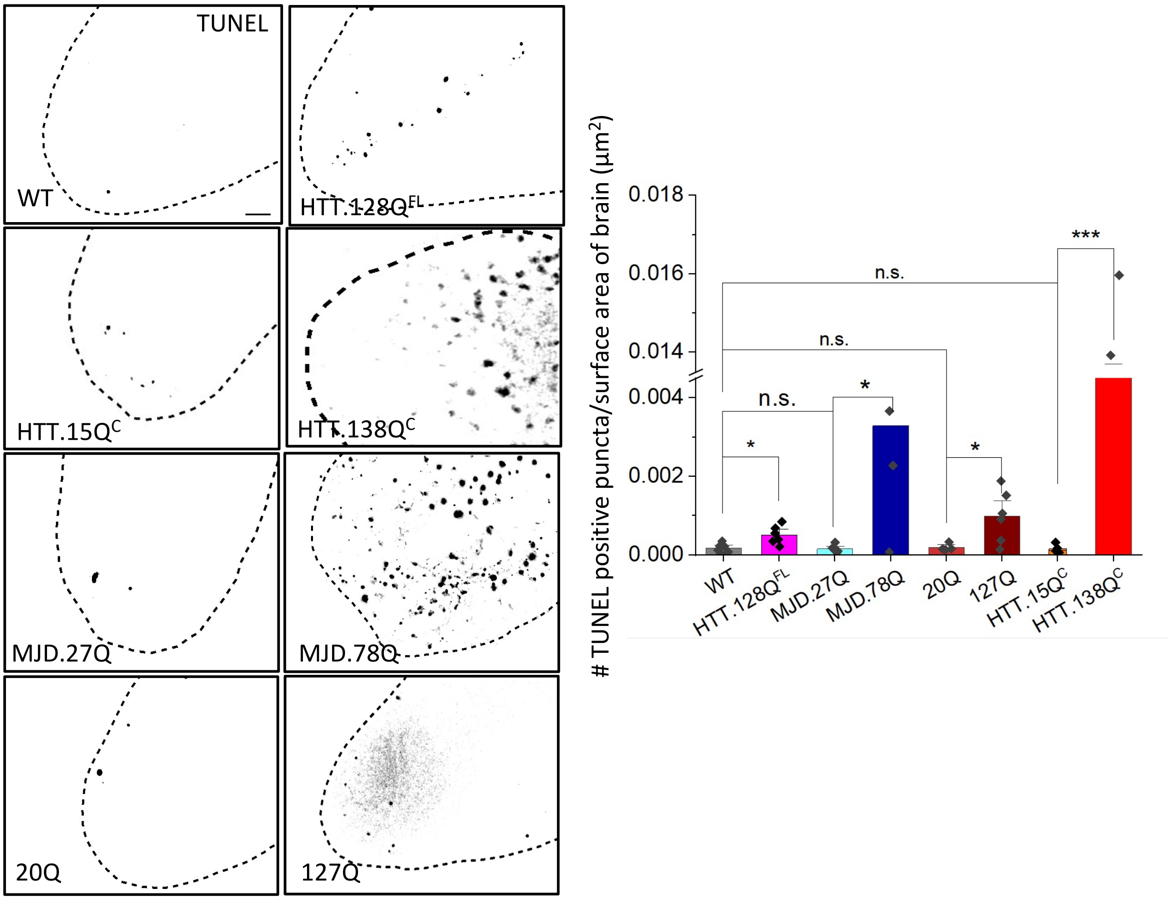

Figure S3. Pathogenic polyQ repeats cause cell death. Representative images from HTT.15Q^{C}, HTT.128Q^{FL}, HTT.138^{C}, MJD.27Q, MJD.78Q, 20Q and 127Q larval brains subjected to the TUNEL assay. Quantification of the number of TUNEL positive cells per surface area (μm^2) from HTT.128Q^{FL} ($p=0.015$), MJD.78Q ($p=0.032$), 127Q ($p=0.031$) and HTT.138^{C} ($p=0.0003$) brains compared to WT, MJD.27Q, 20Q or HTT.15Q^{C}. n=5 larvae per genotype. Scale bar=5μm. Statistical significance was determined using Krushkall-Wallis test followed by Mann-Whitney pairwise comparisons. ***= $p<0.001$, **=$p<0.01$, *=$p<0.05$, n.s.=$p>0.05$

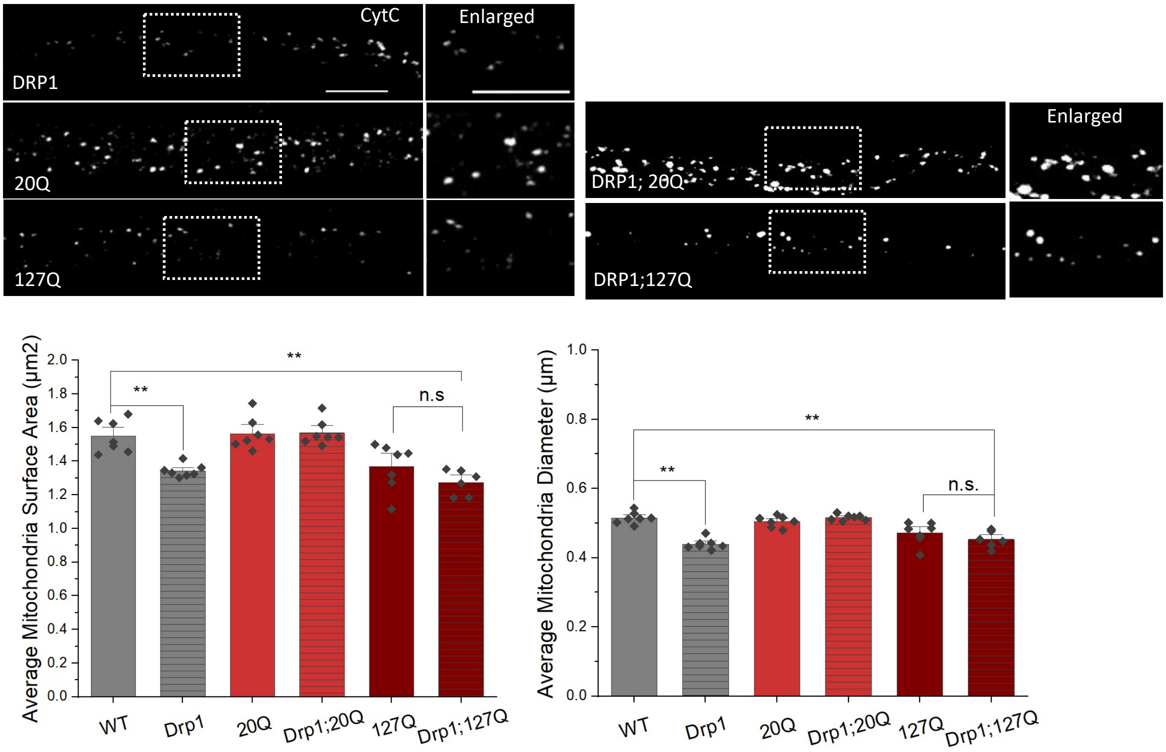

Figure S4. Excess fission protein DRP1 has no effect on polyQ-mediated mitochondrial fragmentation. Representative images from DRP1 expressing larvae or larvae co-expressing DRP1 with 20Q (DRP1;20Q) or with 127Q (DRP1;127Q) stained with CytC. Box shows enlarged area. Quantitative analysis of the average mitochondrial surface area (µm^2) in DRP1;127Q larvae compared to WT larvae (p=0.002). Quantification of the average mitochondrial diameter (µm) in DRP1;127Q larvae compared to WT larvae (p=0.002). Quantification analysis of the average mitochondrial surface area (p=0.003) and diameter (p=0.002) in DRP1 larvae compared to WT. n=6 larvae per genotype. Scale bar=5µm. Statistical significance was determined using Krushkall-Wallis test followed by Mann-Whitney pairwise comparisons. *=p<0.05, **=p<0.01, ***=p<0.001. n.s.=p>0.05

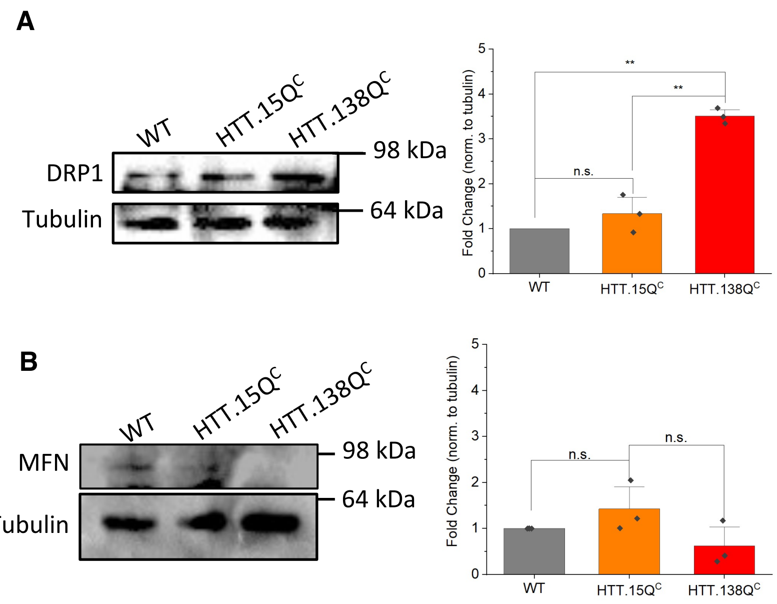

Figure S5. Western blot analysis of DRP1 and MFN levels in pathogenic polyQ larval brains. (A) A representative western blot of proteins isolated from WT, HTT.15Q^{C} and HTT.138Q^{C} larval brains stained with DRP1 and Tubulin (loading control) antibodies. Quantification of the fold change of DRP1 normalized to tubulin in HTT.138^{C} brains compared to WT ($p=0.0015$) or compared to HTT.15Q^{C} ($p=0.0057$). (**B**) A representative western blot of proteins isolated from WT, HTT.15Q^{C} and HTT.138Q^{C} larval brains stained with MFN and Tubulin (loading control) antibodies. Quantification of the fold change of MFN normalized to tubulin in HTT.138^{C} brains compared to WT or to HTT.15Q^{C}. n=3 experiments. Statistical significance was determined via one-way ANOVA followed by Student's t-test. **=$p<0.01$, n.s.=$p>0.05$

Table S1- Comparative summary of HD/polyQ and TBI/mechanical stress conditions

		Wild Type	HD/polyQ expression	TBI/mechanical stress
Fragmented mitochondria	CytC	NO	YES	YES
	MitoTimer	NO	YES	-
	MItoroGFP2-GRX1	NO	YES	-
	MitoroGFP2-Orp1	NO	YES	-
$\Delta\Psi_m$	JC-1	NO	NO	Hyperpolarized
	TMRM	NO	NO	Hyperpolarized
Axonal transport defects	CSP	NO	YES	YES
Cell death	TUNEL	NO	YES	YES
Nitric Oxide	DAF	NO	Increased	n/a
	L-NAME	NO	Rescued fragmented mitochondria	n/a
Fission-Fusion protein	DRP1 Reduction (DRP mutant and mDivi)	-	Rescued fragmented mitochondria	n/a
	DRP1 Overexpression	-	No effect	n/a
	MFN Overexpression	-	Rescued fragmented mitochondria	n/a
